# Epipodial Tentacle Gene Expression and Predetermined Resilience to Summer Mortality in the Commercially Important Greenlip Abalone, *Haliotis laevigata*

**DOI:** 10.1007/s10126-017-9742-z

**Published:** 2017-03-27

**Authors:** Brett P. Shiel, Nathan E. Hall, Ira R. Cooke, Nicholas A. Robinson, Jan M. Strugnell

**Affiliations:** 1 Department of Ecology, Environment and Evolution, School of Life Sciences, La Trobe University, Kingsbury Drive, Melbourne, VIC 3086 Australia; 2 Life Sciences Computation Centre, VLSCI, Parkville, VIC Australia; 3 Department of Molecular and Cell Biology, James Cook University, Townsville, Australia; 4 Department of Biochemistry, La Trobe Institute for Molecular Science, La Trobe University, Kingsbury Drive, Melbourne, VIC 3086 Australia; 5 Nofima, P.O. Box 210, 1431 Ås, Norway; 6 Sustainable Aquaculture Laboratory—Temperate and Tropical (SALTT), School of BioSciences, The University of Melbourne, Parkville, VIC 3010 Australia; 7 Centre for Sustainable Tropical Fisheries and Aquaculture, College of Science and Engineering, James Cook University, Townsville, QLD 4811 Australia

**Keywords:** Differential gene expression, RNA-seq, Heat stress, Aquaculture, Transcriptomics, Mollusk, Abalone

## Abstract

“Summer mortality” is a phenomenon that occurs during warm water temperature spikes that results in the mass mortality of many ecologically and economically important mollusks such as abalone. This study aimed to determine whether the baseline gene expression of abalone before a laboratory-induced summer mortality event was associated with resilience to summer mortality. Tentacle transcriptomes of 35 greenlip abalone (*Haliotis laevigata*) were sequenced prior to the animals being exposed to an increase in water temperature—simulating conditions which have previously resulted in summer mortality. Abalone derived from three source locations with different environmental conditions were categorized as susceptible or resistant to summer mortality depending on whether they died or survived after the water temperature was increased. We detected two genes showing significantly higher expression in resilient abalone relative to susceptible abalone prior to the laboratory-induced summer mortality event. One of these genes was annotated through the NCBI non-redundant protein database using BLASTX to an anemone (*Exaiptasia pallida*) Transposon Ty3-G Gag Pol polyprotein. Distinct gene expression signatures were also found between resilient and susceptible abalone depending on the population origin, which may suggest divergence in local adaptation mechanisms for resilience. Many of these genes have been suggested to be involved in antioxidant and immune-related functions. The identification of these genes and their functional roles have enhanced our understanding of processes that may contribute to summer mortality in abalone. Our study supports the hypothesis that prestress gene expression signatures are indicative of the likelihood of summer mortality.

**Electronic supplementary material:**

The online version of this article (doi:10.1007/s10126-017-9742-z) contains supplementary material, which is available to authorized users.

## Introduction

Over the past few decades, mass mortality of economically important mollusks, such as abalone (Vandepeer 2006), oysters (Cotter et al. 2010), mussels (Mallet et al. 1990), and scallops (Xiao et al. 2005), has been reported during the summer months at a range of locations around the world. These events are known as “summer mortalities” and they occur in both wild and aquaculture environments. During the summer of 2011, water surface temperatures along the coast of Western Australia were recorded to be more than 3 °C above the long-term monthly average (Pearce and Feng 2011). This event resulted in widespread mortality across many species in coastal areas, with Roe’s abalone (*Haliotis roei*) being the most adversely affected species in the area. Mass mortalities of *H. roei* occurred along the entire coast, with many populations reported to be completely wiped out due to the prolonged heat stress (Pearce and Feng 2011).

High water temperature is a key driver of summer mortality and has been linked with outbreaks of infections such as the *Vibrio* sp. bacteria (Romalde et al. 2014; Vezzulli et al. 2010) and the oyster herpes virus (Dégremont 2011) which are known to play a contributing role in these mass mortalities. *Vibrio harveyi*, an infection common to abalone, has recently been shown to have a fast-acting immunosuppressive effect, reducing the effective response of hemocytes in the European abalone *H. tuberculata* (Cardinaud et al. 2015). Higher water temperatures are known to result in chemical changes in water quality (elevated ammonia, changes to pH, lower dissolved oxygen, and nutritional factors) which compromise the immune system and make species more vulnerable to mass mortality from diseases such as those caused by *Vibrio* sp. (Vandepeer 2006).

An increase of only 1 °C in temperature has been demonstrated to have a highly significant impact on mortalities (Travers et al. 2009), and climate change is therefore expected to lead to an increased frequency and severity of summer mortality. Adaptation of abalone to climate change could be difficult if whole populations are subjected to short high-temperature spikes above their optimal range (Vosloo and Vosloo 2010). The optimal and critical thermal maximum temperatures for growth of *Haliotis laevigata* are 18.3 and 27.5 °C, respectively (Gilroy and Edwards 1998).

The susceptibility of adult mollusks to summer mortality in aquaculture can result in large economic losses due to wasted time and resources. The value (e.g., growth and quality) of surviving animals can also be impaired (Morash and Alter 2015). Age is known to be an important factor for mollusks when investigating their reaction to stressors brought on by climate change (Clark et al. 2013). Adult mollusks are known to be more susceptible to the effects of summer mortality than juveniles (Travers et al. 2009). When exposed to increased temperatures, the survival rate of 2-year-old *H. laevigata* has been demonstrated to be significantly higher than when compared to 3-year-old *H. laevigata* (Stone et al. 2014). The timing and energetic requirements of reproduction are suggested to be important factors in determining resistance to summer mortality, with resistant oyster lines known to possess slow gametogenesis and diminished reproductive potential (Huvet et al. 2010). By studying the differential gene expression patterns of the gonads between summer mortality resilient and susceptible Pacific oyster (*Crassostrea gigas*), it has been suggested that the need to invest in reproduction may be resulting in energy trade-offs from important processes such as antioxidant defense, which can result in oxidative stress and mortality (Fleury et al. 2010).

Investigation into resistance to summer mortality has determined that 45% of the variance between the resilient and susceptible strains observed occurs between families (Dégremont et al. 2005) with high heritability (Dégremont et al. 2007). As a result, selective breeding has been considered an effective method to reduce the losses caused by summer mortality. However, due to the complex dynamics of summer mortalities, selection of breeding stocks cannot be simply based on field trials.

A detailed description of molecular and cellular events leading to summer mortality is unavailable due to the complexities and speed of the phenomenon. Molecular methods have the potential to illuminate the basis of summer mortality and explore how the increase in temperature affects the physiology of mollusks. High-throughput genomic approaches have been instrumental in improving knowledge on the genetic basis of resistance to stressors (Maor-Landaw et al. 2014; Zinta et al. 2014) and infectious diseases (Caboche et al. 2014). Sequencing of the transcriptome allows for determination of gene expression signatures, which can then be used as markers for physiological status and health.

“Frontloading” (Barshis et al. 2013) or “preparative defense” (Dong et al. 2008) are terms recently used to describe genes enabling an individual to maintain physiological health, by providing a faster protein level response during stress. These genes are thought to normally have a higher baseline expression in heat-resilient individuals compared to heat-susceptible under ambient temperature. Several molecular chaperones and antioxidant genes have been identified to be more highly expressed in southern populations of the marine snail, *Chlorostoma funebralis* under ambient temperatures, in comparison to northern populations (Gleason and Burton 2015). The authors suggested that the preadaptation to heat stress contributes to the higher thermal tolerance of southern populations. The specific actions of genes with a lower baseline expression in heat-resilient individuals are unclear. However, Barshis et al. (2013) suggested that they might represent a different type of frontloading, with their limited expression having direct effects on the expression of other vital stress-response genes. To date, there has been no study that has specifically analyzed the gene expression differences between summer mortality-susceptible and summer mortality-resilient abalone preceding a heat spike event.

Differential gene expression has recently been investigated in an attempt to predict oyster resilience and susceptibility to summer mortality in *C. gigas*. Several studies have focused on gene expression in the hemolymph and have identified a range of gene functions involved in the process, including genes involved in immune response, metabolism (Taris et al. 2009), cell death, lysosomal proteolysis, and cellular assembly and organization (Chaney and Gracey 2011). Deteriorating oyster health and associated gene expression changes have also been found to occur weeks rather than days before the summer mortality event (Chaney and Gracey 2011). The gene expression differences identified between resilient and susceptible oysters before stress can be maintained after stress, independent of their physical response to bacterial infection (Rosa et al. 2012; Taris et al. 2009). Similar trends have also been identified when utilizing the gonad tissue of *C. gigas*, with many of the differentially expressed genes identified before a summer mortality event, continuing to differentiate susceptible and resilient lines after the temperature rises (Fleury et al. 2010).

If differences in the level of gene expression before heat stress is applied are found to be heritable, it could be possible to use gene signatures to predict the genetic value of an individual in terms of its level of summer mortality resilience, without subjecting candidate breeding animals to the stress (Robinson et al. 2008). By breeding from unstressed individuals with the highest genetic value within families, it should be possible to achieve higher rates of genetic improvement for this trait than would otherwise be possible. Gene expression signatures could then be used in the future to identify and dispose of spat or juveniles that are more susceptible to summer mortality so that resources are not wasted on their production.

The aim of this study was to characterize the gene expression patterns of abalone resilient and susceptible to summer mortality before any heat stress was applied. By utilizing the gene expression data of abalone taken several months before a summer mortality event, we investigate the potential to identify frontloading or preparative gene signatures indicative of resilient abalone long before an impending stress event. We included abalone sourced from three different populations in order to examine universal and population-specific gene expression patterns. The transcriptional frontloading of stress-related genes found to be associated with the resilience of abalone to summer mortality could be used to develop tests in the future to advise farmers about which animals are more likely to survive and thrive over the summer months and also to inform restocking efforts of wild populations.

## Materials and Methods

### Abalone Source

**Fig. 1:**
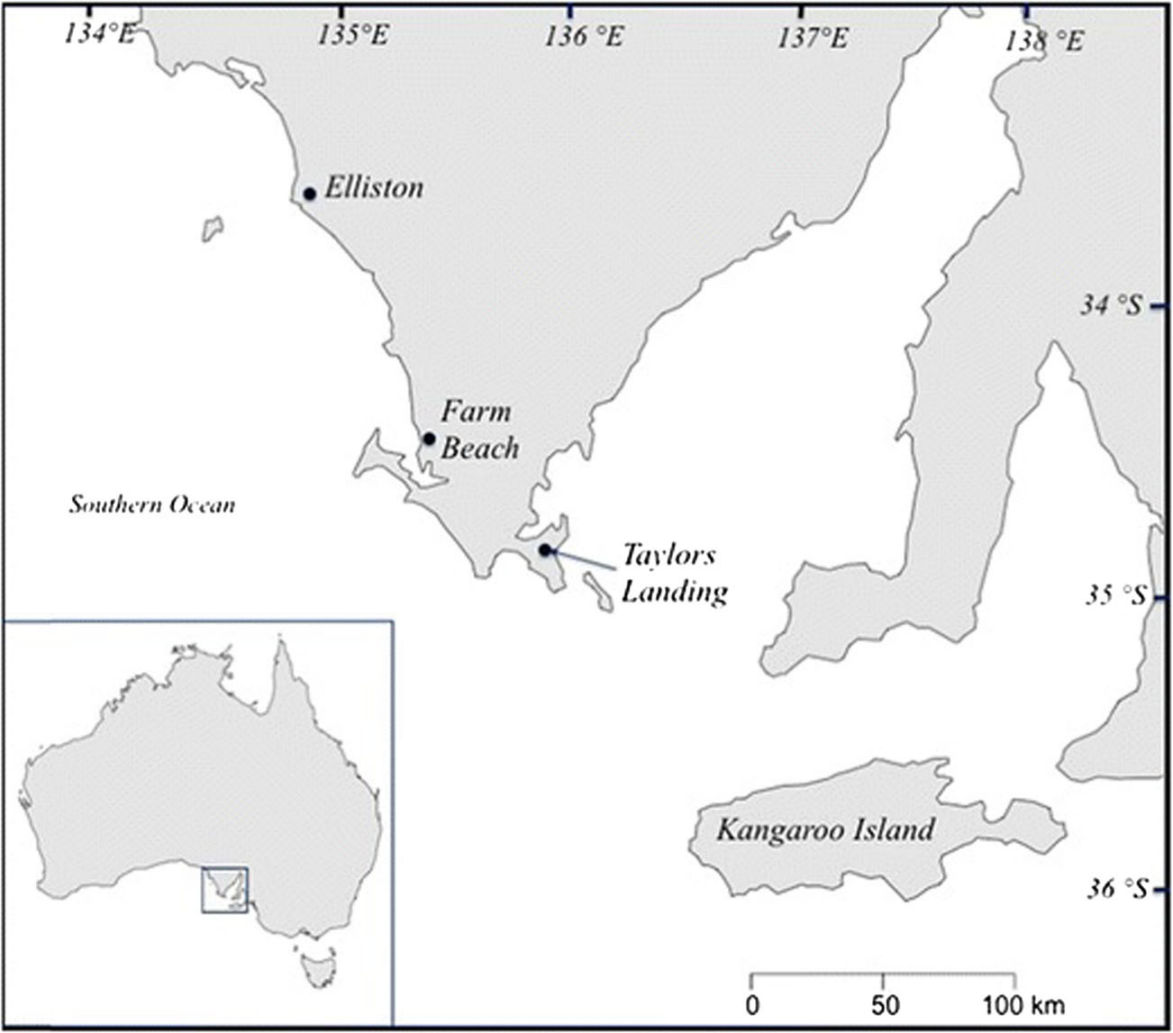
Abalone broodstock sourced from Elliston, Farm Beach, and the Taylor’s Landing and Kangaroo Island region in South Australia, Australia

All abalone used in this study were collected from Southern Australian Seafoods, later Australian Bight Abalone (ABA) in Port Lincoln, South Australia. Ancestry of the abalone used in this study could be traced back at least three generations to wild broodstock sourced originally from Farm Beach (34°24′19.50″S 135°20′52.6″E) and Elliston (33°37′16.53″S 134°50′01.38″E), South Australia (Fig. 1). The broodstock were sourced from reefs of approximately 5- and 30-m depth at Farm Beach and Elliston, respectively. A maximum temperature of 26.8 and 24.5 °C was recorded for Farm Beach and Elliston, respectively, during 23 Nov. 2006–13 Dec. 2007 with data loggers (*Logitech*) positioned nearby (Elliston—33°34′38.8″S, 134°48′24.0″E, water depth 14–20; Farm Beach—34°26′35.2″S, 135°21′20.3″ E, water depth 10–15 m). The reefs at Elliston were comparatively deeper and cooler while those at Farm Beach were shallower and reached higher temperatures. A third group of abalone utilized in this study consisted of animals selectively domesticated based on their faster growth rate by ABA (referred to in this study as the “Aquaculture” population). The origin of these abalone could be traced back to broodstock sourced originally from the region of Taylor’s Landing and Kangaroo Island, South Australia (Robinson et al. 2013) (Fig. 1).

### Sampling and Heat Challenge Test Experiment

An experiment was designed to test summer mortality based on an actual summer mortality event that occurred on farm (Australian Bight Abalone, South Australia) in November 2009, consisting of a sharp increase of 4 °C over a 3-week period which resulted in a significant stock loss. In order to assess whether gene expression patterns prior to heat stress were related to summer mortality resistance, RNA was extracted from abalone 6 months prior to and throughout a heat stress trial. Abalone gene expression was measured a considerable time before the heat stress trial to be able to look for signatures of resilience to summer stress occurring in young abalone long before an impending stress event.

A previous study on oysters utilized a predrilled hole in the shell as a means for inserting a needle and sampling the adductor muscle hemolymph (Wendling et al. 2014). However, abalone are hemophilic and the continuous insertion of needles into an abalone over the trial was expected to have a detrimental effect on abalone health. Tissue and hemolymph from the epipodium have been demonstrated to respond to heat stress through the expression of heat shock proteins in abalone (Liang et al. 2014), therefore epipodial tentacles are deemed an appropriate tissue for the purpose of this transcriptome study. It is likely that epipodial tentacles are bitten or damaged by predators on a regular basis, and thus, it was believed that sampling of these would create minimal stress to animals. Furthermore, the tentacles are believed to have a chemosensory function (Wanichanon et al. 2004), acting as a sensory organ for chemical signals from the environment.

**Fig. 2:**
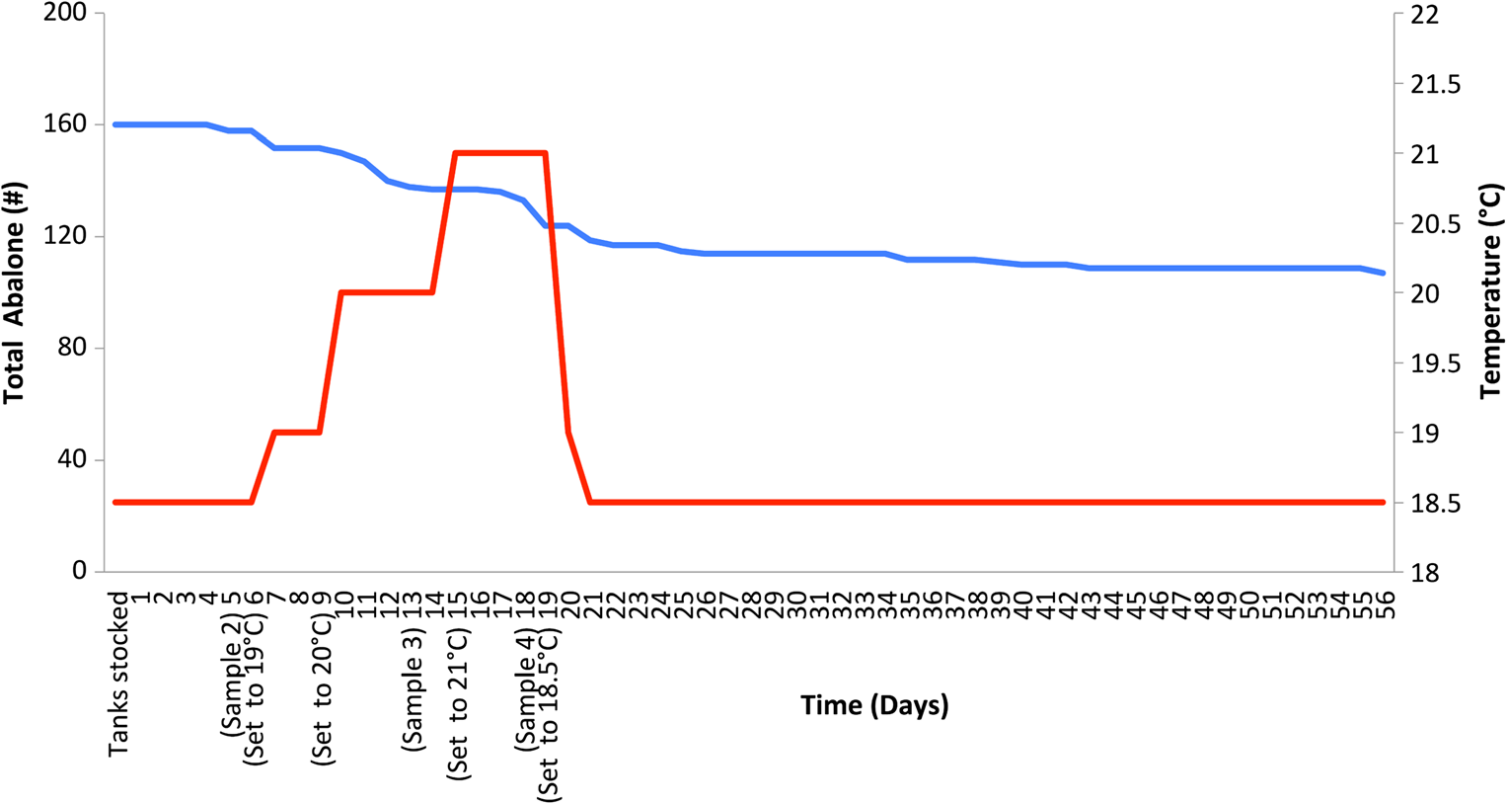
Summer mortality experimental design and result. The *blue line* indicates the number of abalone still alive through experiment. The *red line* indicates the approximate temperature each day through the trial. The x-axis displays the time in days. The trial was run up to day 75, with the last mortality recorded on day 56

In February 2012, at the ABA aquaculture facility, abalone weight and length were measured and an epipodial tentacle sample of each abalone was collected. Abalone used in this trial were spawned in 2009. In June 2012, the abalone were stocked in aquaria at the Port Lincoln Marine Science Centre, South Australia (Fig. 2). Weight and length were measured and abalone were stocked into 16 tanks with a tank capacity of 60 L. Tanks were filled to half capacity, stocked with ten abalone each and acclimatized to conditions at ~18 °C for 5 days. The water quality parameters including temperature and dissolved oxygen were recorded once per day. Abalone health was monitored each day after the acclimatization period over the duration of the experiment. Any abalone recorded to be moribund or dead were removed from aquaria, sampled, and frozen (−80 °C). On day 6, the heaters for each tank were set to 19 °C. Water quality (temperature and dissolved oxygen) was recorded twice per day from this point. On day 9, heaters were raised to 20 °C. On day 18, the heaters were lowered to 18.5 °C following the sample collection. Mortalities throughout the experiment were recorded up to day 75 of the experiment, with the last mortalities recorded on day 56 (Fig. 2). The results from the epipodial tentacle samples taken after temperatures were increased (i.e., on days 5, 13, and 18) will be the subject of further study focusing on differential gene expression during heat stress and are outside the scope of this study. The present study focuses on differential expression prior to heat stress. Abalone that survived the heat trial experiment and remained alive after the 75-day period were deemed to be “resilient.” Abalone that survived after day 18 but died before day 75 were considered to be “susceptible.” Following the rapid increase in temperature and 2 weeks of heat stress, 60 abalone died (deemed susceptible abalone) (Fig. 2), as expected for a summer mortality event.

### RNA Extraction and Sequencing

Abalone selected for RNA sequencing were sourced from six tanks. Each tank contained abalone from multiple populations. The survived/deceased ratio for the Aquaculture, Farm Beach, and Elliston populations in these six tanks was 22:8, 11:5, and 5:6, respectively (57 abalone in total). Three additional abalone in these tanks could not be identified after the heat trial due to tag loss.

Thirty-five abalone from these six tanks were selected for sequencing. These abalone comprised a relatively even number of resilient and susceptible individuals (16 and 19, respectively) and were representative of each of the source locations. The Aquaculture population consisted of seven resilient and seven susceptible abalone. The Elliston population consisted of five resilient and six susceptible abalone, while the Farm Beach population consisted of four resilient and six susceptible abalone. The sex of the abalone was unable to be determined as the animals were immature.

Total RNA was extracted from epipodial tentacle samples collected 6 months prior to stress tests using an RNeasy® Mini Kit (Qiagen) according to the manufacturer’s protocols. Tissue samples were disrupted and homogenized using a desktop homogenizer (*Janke & Kunkel, Ultra-Turrax T25*). RNA quality and quantity was estimated using a *Thermo Scientific Nanodrop 2000*. Quality control and sequencing was outsourced to the Australian Genome Research Facility (AGRF). The quality of the RNA was assessed with an Agilent 2100 Expert Bioanalyzer (Agilent Technologies, Palo Alto, CA, USA), using the Eukaryote Total RNA Nano assay according to the manufacturer’s instructions. All samples had RNA integrity number (RIN) value equal to or greater than 9.6. Library preparation and 100 base pair (bp) single-end RNA sequencing (Illumina HiSeq2000) were conducted on 30 samples (SRA SRP072967). Library preparation and 100 base pair (bp) paired-end RNA sequencing (Illumina HiSeq2000) were conducted on six samples (SRA SRP072967, with R1 used in differential expression analysis). On average, ~9.1 million 100 bp single-end reads per sample were obtained from sequencing. The minimum and maximum read coverage across samples were 6.2 and 13.8 million reads, respectively.

### Sequence Mapping and Differential Expression

In the absence of a reference genome for *H. laevigata*, we generated a de novo assembled transcriptome to use as a reference for read mapping and tentacle gene expression profiling (Shiel et al. 2015). To test for differential gene expression, individual sequence reads for each sample were mapped back to the assembled transcriptome with the alignment program Bowtie (Version 1.0.0) (Langmead et al. 2009) as implemented in Trinity (version 10.5.2012) (Grabherr et al. 2011). Total counts were determined for each gene model by counting the number of reads aligning to each gene model while avoiding multiple counting for reads that mapped to more than one isoform (gene expression sequence data used in this study will be deposited into the NCBI Short-Read Archive under Bioproject PRJNA286263). Count data was then used as input for the R package *EdgeR* (Robinson et al. 2010) which tests for differential expression based on a negative binomial distribution. Tests for differential expression were performed pairwise between different groups of susceptible and resilient abalone. Genes were deemed significantly differentially expressed if they had a Benjamini Hochberg adjusted *P* value (FDR) <0.05 which accounts for multiple testing (Benjamini and Hochberg 2000). Four differential expression analyses were performed to examine the differences in gene expression between susceptible and resilient abalone prior to a summer mortality event: (I) all susceptible vs. all resilient, (II) susceptible vs. resilient Elliston sourced abalone, (III) susceptible vs. resilient Aquaculture sourced abalone, and (IV) susceptible vs. resilient Farm Beach sourced abalone. Weight, length, or growth rate were tested for association with stress resistance, but since no relationship was found, these factors were not included in the analysis model in the interest of parsimony. Family groups (two broad family groups exist for each source location, Online Resource 1: Fig. S1 and S2) and fitness groups (susceptible or resilient) were both included as factors in a general linear model for each location.

$$ \mathrm{Model}=\mathrm{Family}\kern0.5em \mathrm{Group}+\mathrm{Fitness}\kern0.5em \mathrm{Group} $$

These respective analyses examine gene expression differences prior to a summer mortality event under unstressed conditions to identify gene signatures indicative of resilient abalone across all three populations and those that are specific to a single population. A consensus list of differentially expressed genes was then generated from the results of each analysis. When family 1 from Elliston (all offspring of which were recorded as susceptible to summer mortality) was removed from the overall analysis, there was little effect on the results of the analysis (73% of the same genes found to be significantly differentially expressed, FDR < 0.05).

### Functional Annotation and Key Transcript Validation

The abalone transcriptome was annotated using the Trinotate pipeline (version 1.1) (http://trinotate.github.io/). Trinotate provides functional annotations for transcriptome sequences by combining protein prediction (via Transdecoder (http://transdecoder.github.io/) BLAST (Altschul et al. 1990) homology with the UniProt database, identification of Pfam (Finn et al. 2014) domains using HMMER (Finn et al. 2011), prediction of signal peptides using SignalP (Petersen et al. 2011), prediction of transmembrane regions using tmHMM (Krogh et al. 2001), and prediction of rRNA using RNAMMER (Lagesen et al. 2007). SwissProt (Farriol-Mathis et al. 2004) identifiers assigned by Trinotate were used to label differentially expressed genes. If no SwissProt ID was found, genes that could not be assigned a SwissProt ID were annotated using BLASTX (1e-5 threshold) against the *C. gigas* genome (Zhang et al. 2012).

Further annotations of key differentially expressed genes (comp59699 and comp25540) were explored with BLAST searches against non-redundant protein and nucleotide databases. BLASTX results for these sequences were BLAST back to our transcriptome to confirm the alignment. These genes were then validated by aligning reads to the assembly using *Bowtie2* (Langmead and Salzberg 2012) to check for misassembly or large variation in read coverage. These two sequences were also further investigated with a BLASTn “somewhat similar sequence” search for top hit, no E value cut-off.

## Results

### Annotation

**Fig. 3:**
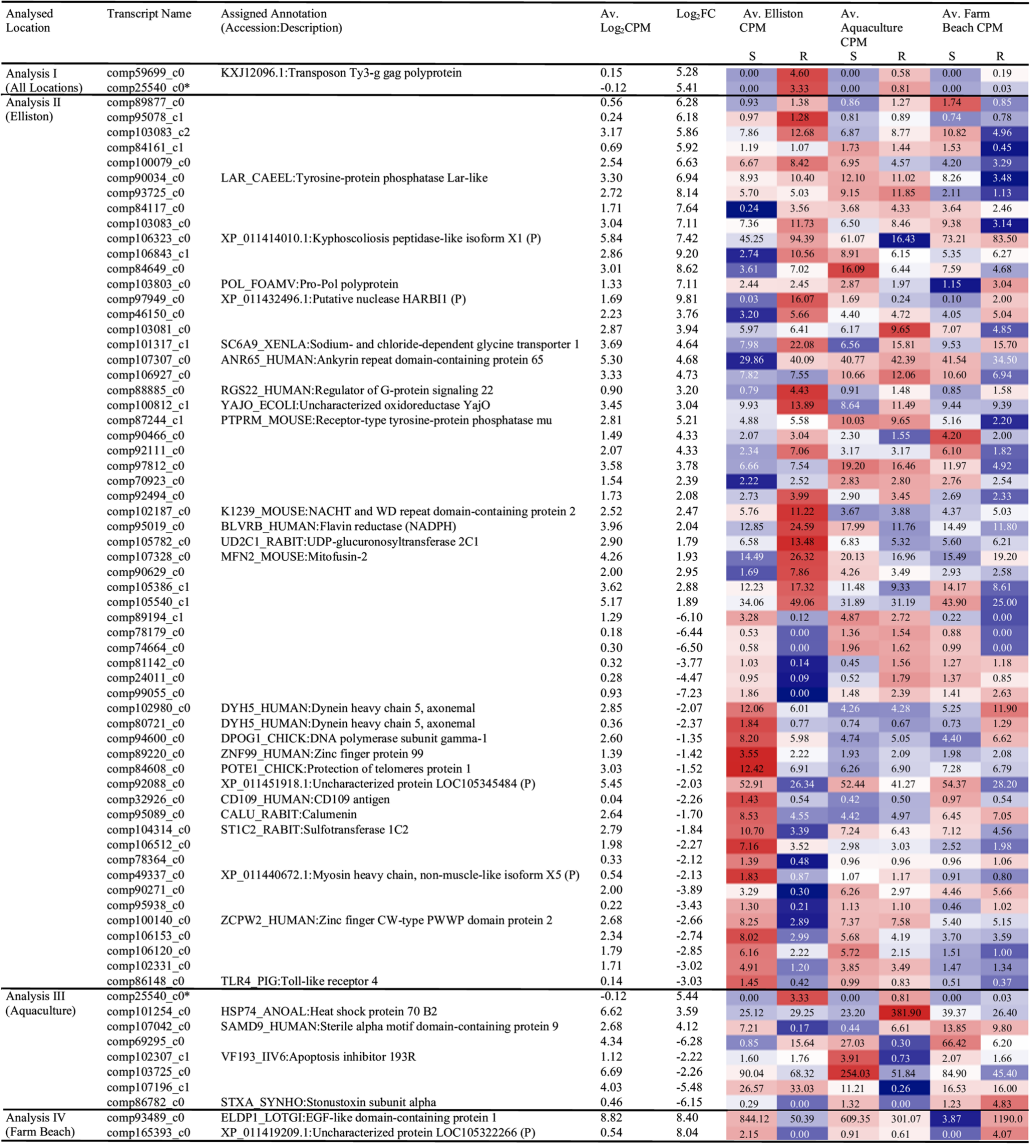
List of differentially expressed genes between susceptible and resilient abalone (FDR < 0.05). Abbreviations: *Log*_*2*_ *CPM* average log counts per million, *Log*_*2*_ *FC* log fold change, *S* susceptible abalone, *R* resilient. Heat map: “*red*” indicates a high average expression, “*white*” indicates moderate average expression, and “*blue*” indicates a low average expression in resilient and susceptible groups. Gene names and descriptions primarily annotated from SwissProt identifiers and secondarily from NCBI using BLASTX (see method). (*) Genes appear in the results of multiple analyses. *P* Predicted BLAST annotations. Av. “*location*” CPM average counts per million of location

Sixty-nine different genes were determined to be differentially expressed in naïve susceptible and resilient abalone (prior to the challenge test), the majority of which were population specific. Of these, 26 genes were matched to known proteins with a Swissprot ID, an additional 5 genes were matched to different known or predicted sequences based on the *C. gigas* genome using BLASTX, and another gene was matched to a similar sequence from the NCBI non-redundant protein database using BLASTX (Fig. 3).

### Gene Signatures for Resistance Detected before the Heat Challenge Test

Differential expression analysis between resilient and susceptible abalone from all locations identified two genes upregulated in resilient abalone prior to the challenge test (Fig. 3). Comp25540 could not be assigned any significant annotation. No similar sequences could be identified in the NCBI non-redundant protein database using BLASTX, regardless of significance cutoff. The most significant hit assigned by BLASTN was to a *H. diversicolor* genomic DNA, BAC clone: 002_c14 (LC027314.1, E value 0.11, query cover 3%, identity 91%, aligned region length 3063–3097). Only one of these genes (comp25540) was also significantly differentially expressed in an analysis of a specific location, that of the “Aquaculture” population. The second gene identified (comp59699_c0) was assigned as a transposon Ty3-g Gag polyprotein (KXJ12096.1, E value: 2e-06, query cover 35%, identity 35%, aligned region length 780–898) gene from the anemone *Exaiptasia pallida* (LogFC 5.28, *FDR* = 2.48E−03). The most significant hit assigned by BLASTN was to a *H. diversicolor* genomic DNA, BAC clone 006_rep_c2415 (LC027343.1, E value 4E−07, query cover 12%, identity 71%, aligned region length 3395–3533, 3781–3895, 3282–3330).

Overall, the gene signatures associated with resilience to the heat challenge showed more differences than similarities in the three sampled populations. Two genes were found to be upregulated in resilient abalone with ancestors derived from Farm Beach (warm water location). One showed some similarity to an uncharacterized protein from *C. gigas* (Log_2_FC 8.04, *FDR =* 7.73E−03), and the other was identified as an EGF-like domain and was significantly upregulated in resilient abalone (Log_2_FC 8.40, *FDR* = 7.73E−03). This gene had the highest read count out of the 69 differentially expressed genes and was also recorded to have one of the highest fold change differences between susceptible and resilient abalone in this study (Fig. 3). The two significant differentially expressed genes identified from Farm Beach were not significant in the two other populations.

Eight differentially expressed genes were identified among abalone with ancestors sourced from the Aquaculture population (Fig. 3). Three genes were found to be upregulated and five were found to be downregulated in resilient abalone. Four of these genes could be annotated. Heat shock protein 70 B2 (HSP74) (Log_2_FC 3.59, *FDR* = 4.24E−02) and the sterile alpha motif domain (SAMD9) (Log_2_FC 4.12, *FDR* = 1.18E−03) genes were significantly upregulated in resilient abalone. SAMD9 had no significant expression pattern difference in both Farm Beach and the Aquaculture populations. An apoptosis inhibitor (VF193) gene was found to be downregulated in resilient Aquaculture abalone (Log_2_FC −2.22, *FDR* = 1.44E−02) (Fig. 3).

Thirty-four genes were found to be upregulated and 25 downregulated in resilient abalone whose ancestors were sourced from Elliston. Twenty-five of the 59 differentially expressed genes identified in the Elliston population could be annotated. Thirteen of these 25 were upregulated and 12 were downregulated in resilient abalone (Fig. 3). The Ankyrin repeats (Log_2_FC 4.68, *FDR* = 4.36E−07), sodium and chloride-dependent glycine transporter (Log_2_FC 4.64, *FDR* = 4.75E−02), and the G-protein signaling-related gene (Log_2_FC 3.20 *FDR* = 2.29E−02) were all significantly upregulated in the resilient Elliston abalone. Although the pattern was weaker and not significant in the Aquaculture and Farm Beach populations, the gene signature patterns were relatively consistent across resilient abalone (Fig. 3). The flavin reductase (NADPH) (Log_2_FC 2.04, *FDR* = 3.37E−02), glucuronosyltransferase (Log_2_FC 1.79, *FDR* = 3.84E−02), and HARBI1-related gene (Log_2_FC 9.81, *FDR* = 4.75E−02) were also all upregulated in resilient Elliston abalone. The Farm Beach analysis suggested the same trend toward upregulation in resilient abalone for these genes; however, the Aquaculture population shows the opposite trend (upregulation in susceptible abalone). The kyphoscoliosis peptidase isoform was significantly upregulated in resilient abalone from Elliston (Log_2_FC 7.42 *FDR =* 1.32E−02). Genes associated with two types of dynein heavy chains, and the gene related to the protection of telomeres had reduced expression in resilient Elliston abalone ([Log_2_FC −2.07, *FDR* = 2.88E−02], [Log_2_FC −2.37, *FDR* = 1.10E−02], [Log_2_FC −1.52, *FDR* = 4.67E−03] respectively); however, the opposite trend was observed for Farm Beach, where upregulation was detected in resilient abalone. CD109 antigen (Log_2_FC −2.26, *FDR* = 4.75E−02), calumenin (Log_2_FC −1.70, *FDR* = 1.95E−02), sulfotransferase (Log_2_FC −1.84, *FDR* = 1.26E−02), and the Toll-like receptor gene (Log_2_FC −3.03, *FDR* = 3.63E−02) were all downregulated in resilient Elliston abalone. Similar gene trends were found in both Aquaculture and Farm Beach populations; however, they were not significant (Fig. 3).

### Family Effect

**Fig. 4:**
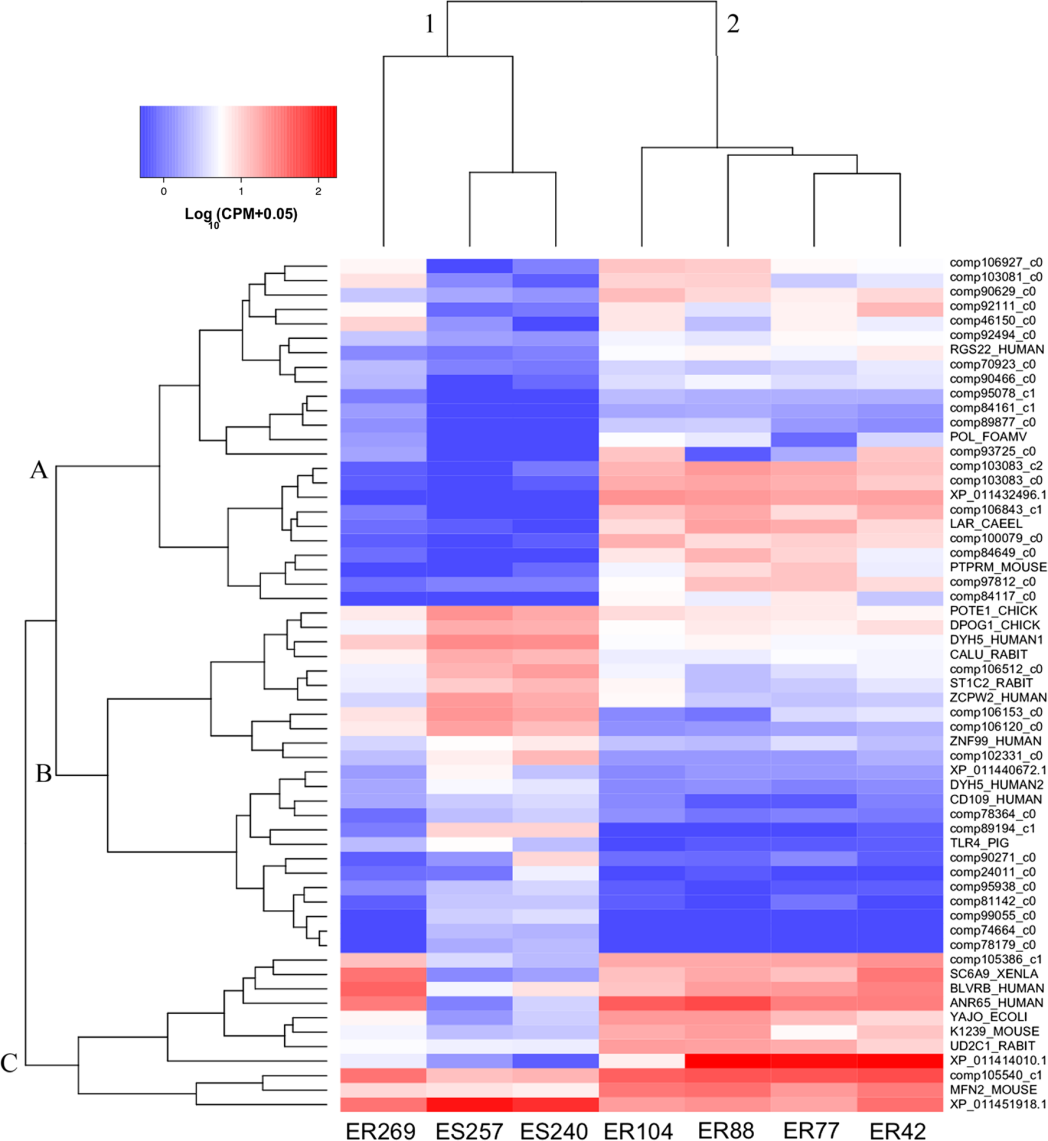
Heatmap of the 59 significantly differentially expressed genes between susceptible and resilient abalone from a closely related group with Elliston ancestry (Online Resource 1: Fig. S1 and S2). Gene clades: (A) and (C) up-regulated in resilient abalone and (B) downregulated in resilient abalone. Tree labels indicate direct family sibling groups: (1) three direct siblings and (2) three direct siblings and one-half sibling (tag no. 42). Abalone labels are indicated on the bottom axis indicating condition (*S* susceptible, *R* resilient) and abalone ID. Family relationships of all individuals are indicated in Online Resource 1

**Fig. 5:**
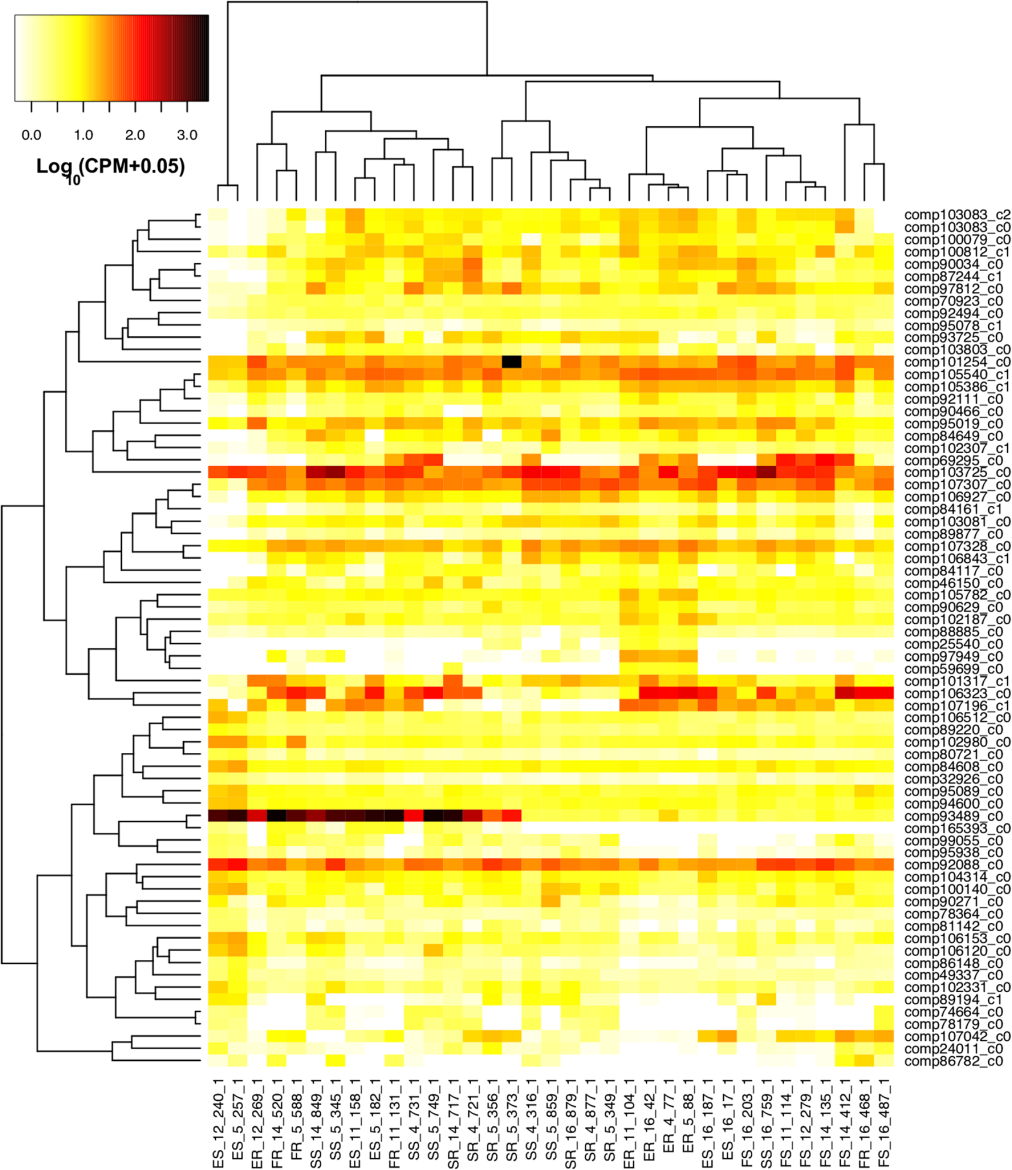
Gene expression heatmap of all 69 significantly differentially expressed genes for all abalone. Abalone labels are indicated on the bottom axis indicating location (*S* Aquaculture, *E* Elliston, *F* Farm Beach), condition (*S* susceptible, *R* resilient), and tank no. during experimental heat trial and abalone ID

To gain insight into the specific patterns in gene expression occurring at a closely related family level, we investigated gene expression signatures of a small group with ancestry to the Elliston population (Fig. 4). Detailed gene expression signature patterns are difficult to interpret when displayed across all samples and differentially expressed genes at once (Fig. 5). Comparing the resilient individuals within these closely related abalone identifies groups of genes that appear to correlate with whether an individual may be resilient or susceptible to the effects of summer mortality (Fig. 4). Genes in clades B and C demonstrated similar gene expression patterns between the resilient individual (R269) (in clade 1) and its resilient cousins in clade 2. Notably, the susceptible siblings had a different gene expression profile to their resilient sibling R269. For example, differential gene expression is observed between susceptible and resilient abalone in genes related to ankyrin repeat domains (ANR65 HUMAN) and sodium and chloride-dependent glycine transporters (SC6A9 XENLA). These two genes are significantly upregulated in resilient compared to susceptible abalone. In direct siblings, the expression pattern of these two genes appears to be representative of resilient and susceptibility. Furthermore, these two genes possessed a similar gene signature pattern (upregulation in resilient abalone) across both the Aquaculture and Farm Beach populations. However, the differential expression within these two populations was not significant (Fig. 3).

## Discussion

Across all populations, two genes were found to be differentially expressed between summer mortality resilient and susceptible abalone 6 months before being subjected to a heat stress event. Both of these genes were significantly upregulated in resilient abalone in comparison to susceptible abalone. One of these genes was annotated as the transposon Ty3-G Gag Pol polyprotein based on homology to a protein from the anemone *E. pallida*. When each population was analyzed separately, we identified 68 genes that were each differentially expressed between resilient and susceptible abalone for particular source locations. These genes could potentially be responsible for mounting preparative defenses prior to stress exposure and summer mortality or potentially resulting in the activation of cascading downstream molecular pathways. These gene signatures may also reflect historical environmental conditions. Tissue and hemolymph from the epipodium has been used by previous studies to measure the transcrptomic response of abalone exposed to temperature stress (Liang et al. 2014). The presence of differentially expressed genes identified in our study between heat stress-resilient and heat stress-susceptible abalone also suggests that the epipodium tissue, specifically that of the tentacles, is a valid tissue to use for transcriptomic analysis of heat-stressed abalone.

### Universal Genes Upregulated and Predetermined Summer Mortality Resilience

When all resilient and susceptible abalone were analyzed together, two differentially expressed genes were identified that were significantly upregulated in resilient abalone before exposure to stress. One of these genes could not be annotated in the NCBI non-redundant protein database using BLASTX (no E-value cutoff). This suggests that this gene could be abalone specific, and as yet unknown due to the abalone being a non-model organism. The second gene identified in this analysis was assigned a significant match to a transposon Ty3-G Gag-Pol polyprotein gene in the anemone (*E. pallida*). Although this transcript shares a region of around 140 AA with a sea anemone transposon, direct homology with a confirmed Ty3-G Gag-pol protein could not be established. It is difficult to say for sure that it is a transposon without access to a *H. laevigata* genome. The transcript has no clear dominant reading frame and has stop codons in the same frame as the Ty3-G blast alignment. This suggests that while it may still be associated with transposon activity, its precise function is unlikely to be the same as Ty3-G. Transposable elements are capable of altering the function of genes with which they become associated through insertion into different positions in the genome. Transposable elements have also been suggested to have a role in epigenetic and adaptive response to environment and stress (Slotkin and Martienssen 2007). No information is currently available for the function of transposable elements in abalone. Similar transposable elements have been shown to be induced by environmental stressors including temperature in the black tiger shrimp (*Penaeus monodon*) and were suggested to have stress-specific regulation and have the potential to be used as effective biomarkers (de la Vega et al. 2007). However, no specific mutagenic activity or function of these elements could be suggested without further sequencing or characterization (de la Vega et al. 2007). Even if these elements do not result in direct transposition, they may still have a significant effect on the physiological status by affecting the regulation of surrounding genes (Krasnov et al. 2005). The Ty3 gene pathway has been studied in many taxa including yeast (Menees and Sandmeyer 1996), mammals (Volff 2009), fungi, plants, insects (Miller et al. 1999), and fish and starfish (Britten et al. 1995). The Ty3 pathway transposition has the potential to dramatically alter gene function and genome structure (Kumar and Bennetzen 1999). The dose and duration of heat and copper stress is positively correlated with the ability of such transposable elements to reshape the genome of fungal plant pathogens, leading to adaptation (Chadha and Sharma 2014).

The sets of differentially expressed genes between susceptible and resilient abalone were distinct between locations/populations when they were analyzed separately. The functions of these genes vary, but all genes that could be identified appear to have a basic function that may be relevant to stress resilience. The ankyrin repeats, sodium and chloride-dependent glycine transporter, and G-protein signaling-related genes were all significantly upregulated in resilient Elliston abalone, with a similar trend found for the other two populations. A similar gene to the sodium and chloride-dependent glycine transporter was found to be differentially expressed in the Pacific oyster (*C. gigas*) in low-salinity environments and has been suggested to be involved in hypo-osmotic adaptation (Meng et al. 2013). Proteins that contain ankyrin repeats are sometimes known to be associated with immune responses (Voronin and Kiseleva 2008), and genes involved with the regulation of G-protein signaling pathways play a major role in cellular responses to extracellular stimuli (De Vries et al. 2000). The naturally high expression of such genes in resilient abalone in comparison to susceptible abalone may play a role in enhancing preparatory defense mechanisms (Dong et al. 2008), potentially enabling the resilient abalone to respond, cope with, or recover from the sudden stress brought on by a sharp increase in water temperature.

### Universal Genes Downregulated and Predetermined Summer Mortality Resilience

The specific actions of genes with a lower baseline expression in heat stress-resistant individuals are not well studied. CD109 antigen, calumenin, sulfotransferase, and Toll-like receptor genes were all significantly downregulated in resilient Elliston abalone, with the same general pattern seen in both Aquaculture and Farm Beach populations. Similar to our findings, high expression of a CD109 antigen precursor has previously been found to be associated with *C. gigas* susceptible to summer mortality (Fleury et al. 2010). The expression pattern of Toll-like receptor genes is triggered by the recognition of microbial signal transduction pathways, assisting the innate immune system in detecting the invasion of microbial pathogens (Takeda and Akira 2005). Calumenin genes play a role in homeostasis, particularly in endoplasmic reticulum stress alleviation, and high expression has been correlated with a significant reduction in apoptosis in stressed cells (Lee and Kwon 2013). The mechanisms regulating the expression of sulfotransferase 1C2 are relatively unknown; however, sulfonation likely plays an important role in the detoxification processes (Gamage et al. 2006). The function of these genes are all related in some way to maintaining cellular health, which suggests that low level of expression of these genes is required to maintain fitness or that these genes are part of a downstream cascade of effects associated with higher resilience. An alternative explanation is that the downregulation of these genes in resilient abalone may allow the upregulation of other vital stress response genes for reducing or fighting stress.

### Population Differences

Considering that this species of abalone possesses a pelagic larval stage of ~1 week (McShane 1992), it is possible that each of these source populations could have low connectivity along the southern coast of Australia. The metapopulation structure of *H. laevigata* has been suggested previously (Brown and Murray 1992). Recently, Miller et al. (2014) studied the connectivity of *H. laevigata* throughout south east Australia and found that there was strong genetic structure (metapopulation structure) throughout the region. They suggested that a 135-km distance was an effective barrier to larval dispersal, with populations largely maintained by self-recruitment within a 30-km area. The three source locations discussed in this study are subjected to different environment conditions. The Farm Beach coast is shallow, usually receiving only very low swell or wind waves, and is sheltered by 100-m-wide sand flats that can produce warmer water. The Elliston coast consists of rocks and reefs with deep colder waters close to the shore. The Aquaculture population had been subjected to an undocumented degree of selection for size and was sourced from regions of the coastline off Kangaroo Island and Taylor’s landings, south Australia. The three original sources of the abalone lines were separated by ~100 km, and, given the geographical spread and topology of the coastline, it is possible that a combination of low gene flow, and/or strong local selection pressures, may be resulting in the distinct gene signatures identified in this study. It is also possible that long-term adaptation to the environmental conditions of origin may have been due to epigenetic effects, whereby environmental stimuli changes DNA methylation, histone, chromatin, and/or other heritable changes affecting gene expression, without the underlying DNA sequence being altered (Moghadam et al. 2015).

Research into mussels with differing osmotic resistance capabilities has suggested that the differences between mussels from different regions is more likely the result of local adaptation than just the result of neutral genetic differences between populations (Landes et al. 2015), or that local adaptation at least plays a major role (Riginos and Cunningham 2005). However, it is difficult to determine whether the differences between the sample populations in this study are a result of local adaptation or just genetic differences brought on by isolation and drift. When populations inhabit a heterogeneous environment, selective pressures on a trait such as stress or disease resilience could differ across the species range. However, the detection of local adaptation is difficult when studying gene expression signatures because demographic history (population expansion, division, and bottlenecks), in the absence of natural selection, could also cause differences in gene expression signatures. By utilizing RNA-seq to sequence the mantle transcriptome and conduct a single nucleotide polymorphism (SNP) analysis of the red abalone (*H. rufescens*) from a range of different environments, Wit and Palumbi (2013) showed that the selection of some genes related to hypoxia resistance and response to heat and pathogens did differ between abalone sourced from differing geographic locations as a result of differing selective pressures along the California coast. However, further study of genetic variation in populations along the southern Australian coast would be needed to determine if such selective pressures exist for *H. laevigata*.

Varying levels of connectivity at different spatial scales have been shown to generate variation in disease resistance, with a higher diversity of resistant phenotypes resulting in higher levels of resistance at the population level (Laine et al. 2011). This phenomenon, where spatial connectivity in the metapopulation promotes genetic variability, may provide *H. laevigata* populations with the diverse armory of genotypes needed to survive the impending effects of increased temperature with climate change. Each of the genes that are differentially expressed in the populations in this study may be associated with different functions, and different preparative defense strategies, which benefit each population under specific local conditions.

### Farm Beach Population

Only two differentially expressed genes were identified between susceptible and resilient abalone. The EGF-like domain gene was highly upregulated in resilient abalone and is one of the most highly differentially expressed genes in this study, as well as having the highest read count of all genes differentially expressed. The function of EGF-like domains is not completely understood; however, they have been suggested to play a role in apoptotic cell removal during inflammation (Park et al. 2008). Upregulation of this gene could prepare abalone to better resist or tolerate the stresses associated with the higher summer temperatures encountered in the Farm Beach environment by leading to the rapid removal of dead or dying cellular material.

### Aquaculture Population

The Aquaculture population was originally sourced from a colder water location; however, it was subject to several generations of selection for favorable traits for increased growth in the farm environment. Two identifiable genes (heat-shock protein 70 B2 and the sterile alpha motif domain) were found to be upregulated in resilient abalone. Sterile alpha motif domains are known to be responsible for regulating cell proliferation and apoptosis (Li et al. 2007) and have been indicated to have an inflammatory response to tissue injury (Chefetz et al. 2008). Apoptosis inhibitor 193R was also significantly upregulated in susceptible Aquaculture abalone. In similar studies, apoptosis inhibitors show higher expression when oysters are infected with herpes virus (Segarra et al. 2014), which has coincided with mass mortalities during the summer months (Friedman et al. 2005).

Almost all organisms react to thermal stress by increasing the expression of heat-shock proteins, an evolutionarily conserved mechanism for maintaining cellular homeostasis during inflammatory response, noxious stimuli, toxins, and free radicals (Lindquist 1986). HSP70 has been the focus of many studies involving summer mortality and thermal stress in abalone for several years (Brokordt et al. 2015; Cheng et al. 2007; Farcy et al. 2007; Li et al. 2012). Interestingly, the HSP70 identified in this study only demonstrated a preadaptive signature in the resilient Aquaculture abalone. This may be due to HSPs being energetically expensive with their expression potentially trading off energy allocations for growth and reproduction (Hofmann and Somero 1995; Somero 2002). Li et al. (2012) found that abalone cultured at higher temperatures responded faster and were more sensitive, expressing more HSP70 over time when exposed to high temperature shock

### Elliston Population

Elliston represents a comparatively cool water source population. This location also showed the greatest number of differentially expressed genes between susceptible and resilient abalone. The genes upregulated in resilient abalone are diverse in function and appear to suggest an involvement in toxin or oxidative stress response. NADPH-dependent flavin reductase activity may be required for a response to heat shock and genotoxic stress in animals (Kwasnicka et al. 2003). HARBI1 has been found to be upregulated in fathead minnows (*Pimephales promelas*) under stress and is suggested to be involved in oxidative metabolism, oxidative stress, apoptosis, and immune function processes (Wiseman et al. 2013). Glucuronosyltransferase is heavily involved in the pathway for foreign material removal in the body which includes toxins, pharmaceuticals, and endogenous substances (Goerres et al. 2006). These genes all displayed the same general expression pattern in Farm Beach abalone but did not exhibit the same pattern in the Aquaculture population.

Kyphoscoliosis peptidase-related genes have been associated with muscle growth and muscle hypertrophy (Miao et al. 2015) and were exclusively upregulated in Elliston resilient abalone. Fast growth rates, gonadal development, and spawning are suggested to coincide with susceptibility to summer mortality in young oysters, possibly due to their high physiological and energetic demand (Cotter et al. 2010). These mollusks may be weakened by reproductive activity making them more vulnerable to disease. However, the negative correlation between summer mortality resilience and reproductive effort does not directly imply there are energetic tradeoffs between reproductive effort and resilience, as bacterial and viral pathogens may be the foremost cause of mortalities (Huvet et al. 2010).

Selection for summer mortality-resilient mollusks has not demonstrated to result in an inadvertent selection for slower or faster growth rates in other studies (Dégremont et al. 2010; Dégremont et al. 2007).

Some of the genes found to be upregulated in susceptible Elliston abalone appear to play roles in maintaining cell function under oxidative stress. The upregulation of genes associated with the protection of telomeres in susceptible abalone suggest that telomeres were at risk, with oxidative stress known to cause exponential telomere shortening (Richter and von Zglinicki 2007). Progressive telomere shortening can lead to chromosome instability and the deterioration of cellular function. Dyneins assist in axonemal transport and are critical to maintain axonemal integrity and have been found to be negatively affected by oxidative stress through reactive oxygen species and linked to neurodegenerative diseases (Fang et al. 2012). The upregulation of these genes in susceptible abalone might suggest that these highly expressed axonemal and telomere defense genes may come at an energetic cost to the animal and may contribute to their eventual susceptibility to summer mortality.

## Conclusion

The ability to predict summer mortality susceptibility and resilience appears to be complex as few species-wide or even population-wide gene signatures were detected. A significant outcome of this study is the identification of genes that may highlight important areas of interest for future study, such as transposable elements and their potential role in epigenetic systems. Our data supports the hypothesis that summer mortality resilience could be partly influenced by prestress gene expression differences that prime an animal’s physiology so that it can respond faster and more effectively to summer mortality events. As the analysis of differential expression was performed using a non-lethal method of tentacle sampling, we were able to sample naïve animals before the stress challenge and look for associations between baseline gene expression signatures and resilience or susceptibility to heat stress. This non-lethal method could also allow us to sample, test, and possibly provide the ability to make predictions about the susceptibility of animals before the occurrence of the stress. This would be of significant value to the abalone aquaculture industry. With impending threats such as climate change, the genes identified in this study could also be incorporated into large-scale management programs for coastal systems aimed at conservation and restocking of wild abalone and other summer mortality-affected animals by identifying populations at risk.

## Electronic supplementary material

ESM 1 (DOCX 132 kb)

ESM 2 (DOCX 78 kb)

ESM 3 (DOCX 42 kb)

ESM 4 (DOCX 133 kb)

## References

[CR1] Altschul SF, Gish W, Miller W, Myers EW, Lipman DJ (1990). Basic local alignment search tool. J Mol Biol.

[CR2] Barshis DJ, Ladner JT, Oliver TA, Seneca FO, Traylor-Knowles N, Palumbi SR (2013). Genomic basis for coral resilience to climate change. Proc Natl Acad Sci U S A.

[CR3] Benjamini Y, Hochberg Y (2000). On the adaptive control of the false discovery rate in multiple testing with independent statistics. J Educ Behav Stat.

[CR4] Britten RJ, McCormack TJ, Mears TL, Davidson EH (1995). Gypsy/Ty3-class retrotransposons integrated in the DNA of herring, tunicate, and echinoderms. J Mol Evol.

[CR5] Brokordt KB, González RC, Farías WJ, Winkler FM (2015). Potential response to selection of HSP70 as a component of innate immunity in the abalone *Haliotis rufescens*. PLoS One.

[CR6] Brown L, Murray N (1992) Genetic relationships within the genus *Haliotis*. Abalone of the world: biology, fisheries and culture. Blackwell Scientific Publishers, London, pp 19–23

[CR7] Caboche S, Audebert C, Hot D (2014). high-throughput sequencing, a versatile weapon to support genome-based diagnosis in infectious diseases: applications to Clinical Bacteriology. Pathogens.

[CR8] Cardinaud M, Dheilly NM, Huchette S, Moraga D, Paillard C (2015). The early stages of the immune response of the European abalone *Haliotis tuberculata* to a *Vibrio harveyi* infection. Dev Comp Immunol.

[CR9] Chadha S, Sharma M (2014). Transposable elements as stress adaptive capacitors induce genomic instability in fungal pathogen *Magnaporthe oryzae*. PLoS One.

[CR10] Chaney ML, Gracey AY (2011). Mass mortality in Pacific oysters is associated with a specific gene expression signature. Mol Ecol.

[CR11] Chefetz I (2008). Normophosphatemic familial tumoral calcinosis is caused by deleterious mutations in SAMD9, encoding a TNF-α responsive protein. J Invest Dermatol.

[CR12] Cheng P, Liu X, Zhang G, He J (2007). Cloning and expression analysis of a HSP70 gene from Pacific abalone (*Haliotis discus hannai*). Fish Shellfish Immunol.

[CR13] Clark MS (2013). Hypoxia impacts large adults first: consequences in a warming world. Glob Chang Biol.

[CR14] Cotter E (2010). Summer mortality of the Pacific oyster, *Crassostrea gigas*, in the Irish Sea: the influence of growth, biochemistry and gametogenesis. Aquaculture.

[CR15] de la Vega E, Degnan BM, Hall MR, Wilson KJ (2007). Differential expression of immune-related genes and transposable elements in black tiger shrimp (*Penaeus monodon*) exposed to a range of environmental stressors. Fish Shellfish Immunol.

[CR16] De Vries L, Zheng B, Fischer T, Elenko E, Farquhar MG (2000). The regulator of G protein signaling family. Annu Rev Pharmacol Toxicol.

[CR17] Dégremont L (2011). Evidence of herpes virus (OsHV-1) resistance in juvenile *Crassostrea gigas* selected for high resistance to the summer mortality phenomenon. Aquaculture.

[CR18] Dégremont L (2005). Relative importance of family, site, and field placement timing on survival, growth, and yield of hatchery-produced Pacific oyster spat (*Crassostrea gigas*). Aquaculture.

[CR19] Dégremont L, Ernande B, Bédier E, Boudry P (2007). Summer mortality of hatchery-produced Pacific oyster spat (*Crassostrea gigas*). I. Estimation of genetic parameters for survival and growth. Aquaculture.

[CR20] Dégremont L, Bédier E, Boudry P (2010). Summer mortality of hatchery-produced Pacific oyster spat (*Crassostrea gigas*). II. Response to selection for survival and its influence on growth and yield. Aquaculture.

[CR21] Dong Y, Miller LP, Sanders JG, Somero GN (2008). Heat-shock protein 70 (Hsp70) expression in four limpets of the genus *Lottia*: interspecific variation in constitutive and inducible synthesis correlates with in situ exposure to heat stress. Biol Bull.

[CR22] Fang Y, Tian X, Bai S, Fan J, Hou W, Tong H, Li D (2012). Autologous transplantation of adipose-derived mesenchymal stem cells ameliorates streptozotocin-induced diabetic nephropathy in rats by inhibiting oxidative stress, pro-inflammatory cytokines and the p38 MAPK signaling pathway. Int J Mol Med.

[CR23] Farcy E, Serpentini A, Fiévet B, Lebel J-M (2007). Identification of cDNAs encoding HSP70 and HSP90 in the abalone *Haliotis tuberculata*: transcriptional induction in response to thermal stress in hemocyte primary culture. Comp Biochem Physiol B Biochem Mol Biol.

[CR24] Farriol-Mathis N (2004). Annotation of post-translational modifications in the Swiss-Prot knowledge base. Proteomics.

[CR25] Finn RD, Clements J, Eddy SR (2011). HMMER web server: interactive sequence similarity searching. Nucleic Acids Res.

[CR26] Finn RD et al (2014) Pfam: the protein families database. Nucleic Acids Res 42(Database issue):D222–D230 10.1093/nar/gkt1223 PMC3965110 24288371

[CR27] Fleury E (2010). Microarray-based identification of gonad transcripts differentially expressed between lines of Pacific oyster selected to be resistant or susceptible to summer mortality. Mar Biotechnol.

[CR28] Friedman CS (2005). Herpes virus in juvenile Pacific oysters *Crassostrea gigas* from Tomales Bay, California, coincides with summer mortality episodes. Dis Aquat Org.

[CR29] Gamage N, Barnett A, Hempel N, Duggleby RG, Windmill KF, Martin JL, McManus ME (2006). Human sulfotransferases and their role in chemical metabolism. Toxicol Sci.

[CR30] Gilroy A, Edwards S (1998). Optimum temperature for growth of Australian abalone: preferred temperature and critical thermal maximum for blacklip abalone, *Haliotis rubra* (Leach), and greenlip abalone, *Haliotis laevigata* (Leach). Aquac Res.

[CR31] Gleason LU, Burton RS (2015). RNA-seq reveals regional differences in transcriptome response to heat stress in the marine snail *Chlorostoma funebralis*. Mol Ecol.

[CR32] Goerres M, Roelofs H, Jansen J, Peters W (2006). Deficient UDP-glucuronosyltransferase detoxification enzyme activity in the small intestinal mucosa of patients with coeliac disease. Aliment Pharmacol Ther.

[CR33] Grabherr MG (2011). Full-length transcriptome assembly from RNA-Seq data without a reference genome. Nat Biotechnol.

[CR34] Hofmann G, Somero G (1995). Evidence for protein damage at environmental temperatures: seasonal changes in levels of ubiquitin conjugates and hsp70 in the intertidal mussel *Mytilus trossulus*. J Exp Biol.

[CR35] Huvet A, Normand J, Fleury E, Quillien V, Fabioux C, Boudry P (2010). Reproductive effort of Pacific oysters: a trait associated with susceptibility to summer mortality. Aquaculture.

[CR36] Krasnov A, Koskinen H, Afanasyev S, Mölsä H (2005). Transcribed Tc1-like transposons in salmonid fish. BMC Genomics.

[CR37] Krogh A, Larsson B, Von Heijne G, Sonnhammer EL (2001). Predicting transmembrane protein topology with a hidden Markov model: application to complete genomes. J Mol Biol.

[CR38] Kumar A, Bennetzen JL (1999). Plant retrotransposons. Annu Rev Genet.

[CR39] Kwasnicka DA, Krakowiak A, Thacker C, Brenner C, Vincent SR (2003). Coordinate expression of NADPH-dependent flavin reductase, Fre-1, and Hint-related 7meGMP-directed hydrolase, DCS-1. J Biol Chem.

[CR40] Lagesen K, Hallin P, Rødland EA, Stærfeldt H-H, Rognes T, Ussery DW (2007). RNAmmer: consistent and rapid annotation of ribosomal RNA genes. Nucleic Acids Res.

[CR41] Laine AL, Burdon JJ, Dodds PN, Thrall PH (2011). Spatial variation in disease resistance: from molecules to metapopulations. J Ecol.

[CR42] Landes A, Dolmer P, Poulsen LK, Petersen JK, Vismann B (2015). Growth and respiration in blue mussels (*Mytilus* spp.) from different salinity regimes. J Shellfish Res.

[CR43] Langmead B, Salzberg SL (2012). Fast gapped-read alignment with Bowtie 2. Nat Methods.

[CR44] Langmead B, Trapnell C, Pop M, Salzberg SL (2009). Ultrafast and memory-efficient alignment of short DNA sequences to the human genome. Genome Biol.

[CR45] Lee JH, Kwon EJ (2013). Calumenin has a role in the alleviation of ER stress in neonatal rat cardiomyocytes. Biochem Biophys Res Commun.

[CR46] Li CF (2007). Human sterile alpha motif domain 9, a novel gene identified as down-regulated in aggressive fibromatosis, is absent in the mouse. BMC Genomics.

[CR47] Li J, He Q, Sun H, Liu X (2012). Acclimation-dependent expression of heat shock protein 70 in Pacific abalone (*Haliotis discus hannai* Ino) and its acute response to thermal exposure. Chin J Oceanol Limnol.

[CR48] Liang S, Luo X, You W, Luo L, Ke C (2014). The role of hybridization in improving the immune response and thermal tolerance of abalone. Fish Shellfish Immunol.

[CR49] Lindquist S (1986). The heat-shock response. Annu Rev Biochem.

[CR50] Mallet A, Carver C, Freeman K (1990). Summer mortality of the blue mussel in eastern Canada: spatial, temporal, stock and age variation. Mar Ecol Prog Ser.

[CR51] Maor-Landaw K, Karako-Lampert S, Ben-Asher HW, Goffredo S, Falini G, Dubinsky Z, Levy O (2014). Gene expression profiles during short-term heat stress in the red sea coral *Stylophora pistillata*. Glob Chang Biol.

[CR52] McShane P (1992) Early life history of abalone: a review. Abalone of the world: biology, fisheries and culture. Blackwell Scientific Publishers, London, pp 120–138

[CR53] Menees TM, Sandmeyer SB (1996). Cellular stress inhibits transposition of the yeast retrovirus-like element Ty3 by a ubiquitin-dependent block of virus-like particle formation. Proc Natl Acad Sci.

[CR54] Meng J (2013). Genome and transcriptome analyses provide insight into the euryhaline adaptation mechanism of *Crassostrea gigas*. PLoS One.

[CR55] Miao Y, Yang J, Xu Z, Jing L, Zhao S, Li X (2015). RNA sequencing identifies upregulated kyphoscoliosis peptidase and phosphatidic acid signaling pathways in muscle hypertrophy generated by transgenic expression of myostatin propeptide. Int J Mol Sci.

[CR56] Miller K, Lynch C, Martin J, Herniou E, Tristem M (1999). Identification of multiple Gypsy LTR-retrotransposon lineages in vertebrate genomes. J Mol Evol.

[CR57] Miller K, Mundy C, Mayfield S (2014). Molecular genetics to inform spatial management in benthic invertebrate fisheries: a case study using the Australian Greenlip Abalone. Mol Ecol.

[CR58] Moghadam H, Mørkøre T, Robinson N (2015). Epigenetics—potential for programming fish for aquaculture?. J Mar Sci Eng.

[CR59] Morash AJ, Alter K (2015). Effects of environmental and farm stress on abalone physiology: perspectives for abalone aquaculture in the face of global climate change. Rev Aquac.

[CR60] Park S (2008). Rapid cell corpse clearance by stabilin-2, a membrane phosphatidylserine receptor. Cell Death Differ.

[CR61] Pearce A, Feng M (2011) The “marine heat wave” off Western Australia during the summer of 2010/11. Western Australian Fisheries and Marine Research Laboratories, Fisheries report No. 222, Department of Fisheries

[CR62] Petersen TN, Brunak S, von Heijne G, Nielsen H (2011). SignalP 4.0: discriminating signal peptides from transmembrane regions. Nat Methods.

[CR63] Richter T, von Zglinicki T (2007). A continuous correlation between oxidative stress and telomere shortening in fibroblasts. Exp Gerontol.

[CR64] Riginos C, Cunningham CW (2005). Invited review: local adaptation and species segregation in two mussel (*Mytilus edulis* × *Mytilus trossulus*) hybrid zones. Mol Ecol.

[CR65] Robinson N, Goddard M, Hayes B (2008). Use of gene expression data for predicting continuous phenotypes for animal production and breeding. Animal.

[CR66] Robinson MD, McCarthy DJ, Smyth GK (2010). edgeR: a bioconductor package for differential expression analysis of digital gene expression data. Bioinformatics.

[CR67] Robinson N, Smith B, Cooke I, Strugnell J (2013). A snail's pace: a preliminary analysis of the effects of stress and genetics on movement of *Haliotis*. Aquaculture.

[CR68] Romalde JL, Diéguez AL, Lasa A, Balboa S (2014) New *Vibrio* species associated to molluscan microbiota: a review. Front Microbiol 4:413 10.3389/fmicb.2013.00413 PMC3877837 24427157

[CR69] Rosa RD, De Lorgeril J, Tailliez P, Bruno R, Piquemal D, Bachère E (2012). A hemocyte gene expression signature correlated with predictive capacity of oysters to survive *Vibrio* infections. BMC Genomics.

[CR70] Segarra A, Faury N, Pépin J-F, Renault T (2014). Transcriptomic study of 39 ostreid herpesvirus 1 genes during an experimental infection. J Invertebr Pathol.

[CR71] Shiel BP, Hall NE, Cooke IR, Robinson NA, Strugnell JM (2015). De novo characterisation of the greenlip abalone transcriptome (*Haliotis laevigata*) with a focus on the heat shock protein 70 (HSP70) family. Mar Biotechnol.

[CR72] Slotkin RK, Martienssen R (2007). Transposable elements and the epigenetic regulation of the genome. Nat Rev Genet.

[CR73] Somero GN (2002). Thermal physiology and vertical zonation of intertidal animals: optima, limits, and costs of living. Integr Comp Biol.

[CR74] Stone DA, Bansemer MS, Lange B, Schaefer EN, Howarth GS, Harris JO (2014). Dietary intervention improves the survival of cultured greenlip abalone (*Haliotis laevigata* Donovan) at high water temperature. Aquaculture.

[CR75] Takeda K, Akira S (2005). Toll-like receptors in innate immunity. Int Immunol.

[CR76] Taris N, Lang R, Reno P, Camara M (2009). Transcriptome response of the Pacific oyster (*Crassostrea gigas*) to infection with *Vibrio tubiashii* using cDNA AFLP differential display. Anim Genet.

[CR77] Travers MA, Basuyaux O, Le Goïc N, Huchette S, Nicolas JL, Koken M, Paillard C (2009). Influence of temperature and spawning effort on *Haliotis tuberculata* mortalities caused by *Vibrio harveyi*: an example of emerging vibriosis linked to global warming. Glob Chang Biol.

[CR78] Vandepeer M (2006). Preventing summer mortality of abalone in aquaculture systems by understanding interactions between nutrition and water temperature.

[CR79] Vezzulli L, Previati M, Pruzzo C, Marchese A, Bourne DG, Cerrano C (2010). *Vibrio* infections triggering mass mortality events in a warming Mediterranean Sea. Environ Microbiol.

[CR80] Volff JN (2009). Cellular genes derived from Gypsy/Ty3 retrotransposons in mammalian genomes. Ann N Y Acad Sci.

[CR81] Voronin D, Kiseleva E (2008) Functional role of proteins containing ankyrin repeats. Cell Tiss Biol 2:1–12 18318217

[CR82] Vosloo D, Vosloo A (2010). Response of cold-acclimated, farmed South African abalone (*Haliotis midae*) to short-term and long-term changes in temperature. J Therm Biol.

[CR83] Wanichanon C (2004). Sensory receptors on cephalic and epipodial tentacles of *Haliotis asinina* Linnaeus. J Shellfish Res.

[CR84] Wendling CC, Batista FM, Wegner KM (2014). Persistence, seasonal dynamics and pathogenic potential of *Vibrio* communities from Pacific oyster hemolymph. PLoS One.

[CR85] Wiseman SB, He Y, Gamal-El Din M, Martin JW, Jones PD, Hecker M, Giesy JP (2013). Transcriptional responses of male fathead minnows exposed to oil sands process-affected water. Comp Biochem Physiol C Toxicol Pharmacol.

[CR86] Wit P, Palumbi SR (2013). Transcriptome-wide polymorphisms of red abalone (*Haliotis rufescens*) reveal patterns of gene flow and local adaptation. Mol Ecol.

[CR87] Xiao J, Ford SE, Yang H, Zhang G, Zhang F, Guo X (2005). Studies on mass summer mortality of cultured zhikong scallops (*Chlamys farreri* Jones et Preston) in China. Aquaculture.

[CR88] Zhang G (2012). The oyster genome reveals stress adaptation and complexity of shell formation. Nature.

[CR89] Zinta G (2014). Physiological, biochemical, and genome-wide transcriptional analysis reveals that elevated CO2 mitigates the impact of combined heat wave and drought stress in *Arabidopsis thaliana* at multiple organizational levels. Glob Chang Biol.

