# Supplementary material for: Epipodial Tentacle Gene Expression and Predetermined Resilience to Summer Mortality in the Commercially Important Greenlip Abalone, Haliotis laevigata
Source: Mar Biotechnol (NY). 2017 Mar 27;19(2):191–205. doi: 10.1007/s10126-017-9742-z (PMC5405107; doi:10.1007/s10126-017-9742-z)
Supplement: Supplementary file 1 — (DOCX 132 kb) [file 10126_2017_9742_MOESM1_ESM.docx]

**Article Title:** Epipodial tentacle gene expression and predetermined resilience to summer mortality in the commercially important green lip abalone, *Haliotis laevigata*

**Journal Name:** Marine Biotechnology

**Authors:**

Brett P. Shiel^1^, Nathan E. Hall^1,2,4^, Ira R. Cooke^3,2,4^, Nicholas A. Robinson^5,6^, Jan M. Strugnell^7,1^

1. Department of Ecology, Environment and Evolution, School of Life Sciences, La Trobe University, Kingsbury Drive, Melbourne, Vic. 3086, Australia
2. Life Sciences Computation Centre, VLSCI, Parkville, Vic, Australia
3. Department of Molecular and Cell Biology, James Cook University, Townsville, Australia
4. Department of Biochemistry, La Trobe Institute for Molecular Science, La Trobe University, Kingsbury Drive, Melbourne, Vic. 3086, Australia
5. Nofima, P.O. Box 210, 1431 Ås, Norway.
6. Sustainable Aquaculture Laboratory - Temperate and Tropical (SALTT), School of BioSciences, The University of Melbourne, Parkville, Vic. 3010, Australia
7. Centre for Sustainable Tropical Fisheries & Aquaculture, College of Science and Engineering, James Cook University, Townsville, Qld, 4811, Australia

**Corresponding author:**

Email: [bpshiel@students.latrobe.edu.au](mailto:bpshiel@students.latrobe.edu.au)

Phone: 0417151109

Fax: 03 9479 1266

Postal Address:

Brett Shiel

Department of Ecology, Environment & Evolution

La Trobe University, Plenty Road & Kingsbury Drive, Melbourne VIC 3086, Australia


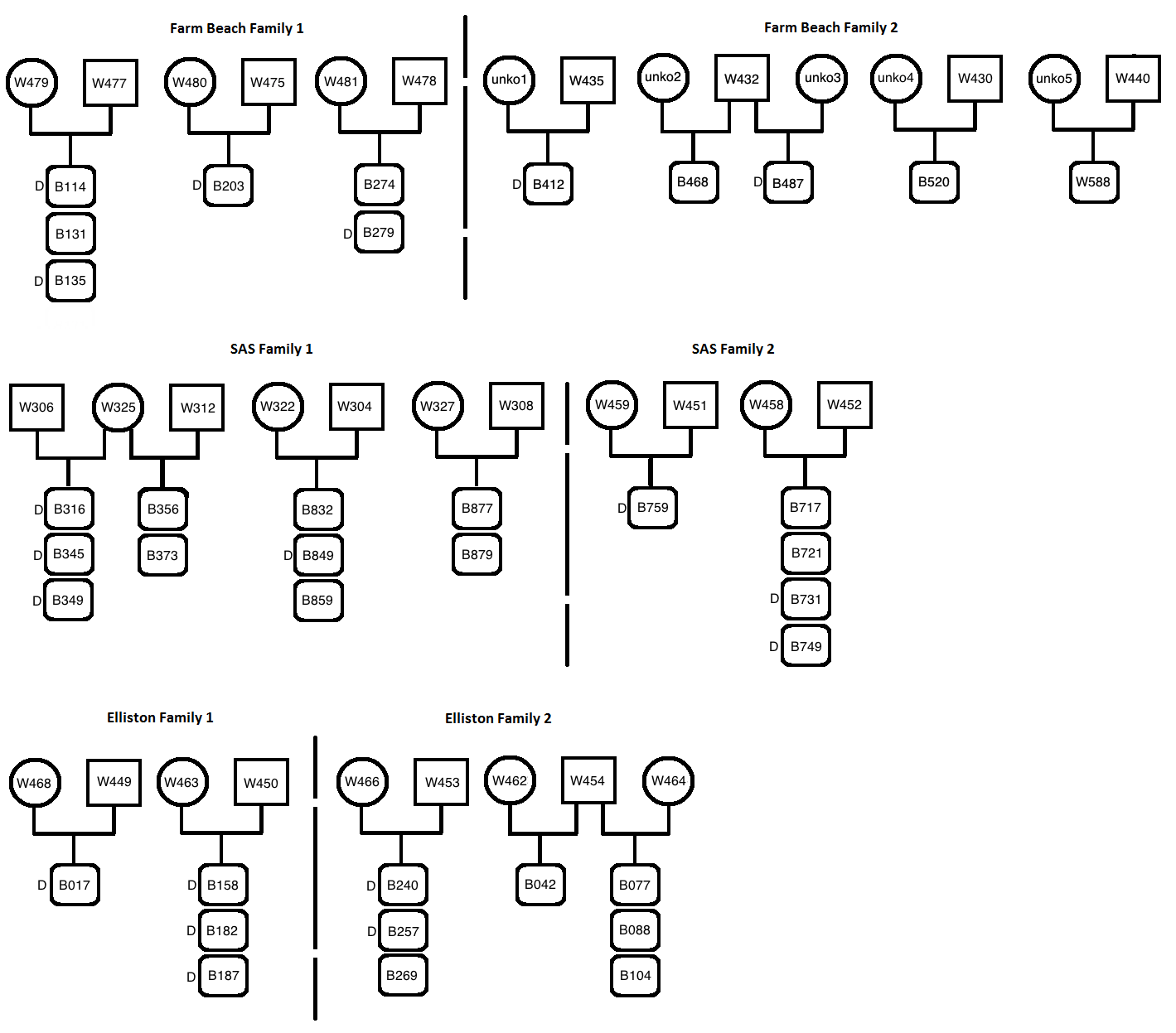
**Online Resource 1: Fig. S1.** Pedigree information and family groups of Elliston, Farm Beach and Aquaculture locations/populations. Elliston and Farm Beach are stock bred in an aquaculture farm environment (ABA) from field-sourced abalone parents. ‘Aquaculture’ family groups have ancestors sourced from Kangaroo Island and the Taylor’s Landing region, South Australia. “D” signifies abalone that died during the heat trial experiment and therefore were deemed susceptible to summer mortality.


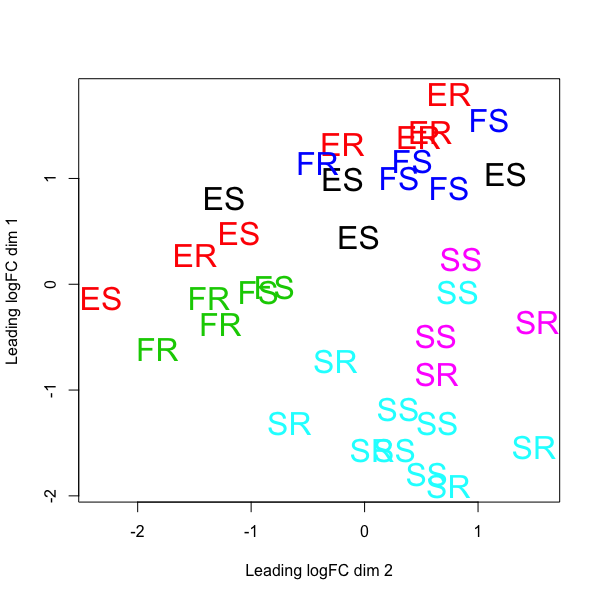


**Online Resource 1: Fig. S2.** MDS plot of family groups of Elliston, Farm Beach and Aquaculture populations with the top 200 expressed genes. Elliston and Farm Beach are stock bred in an aquaculture farm environment (ABA) from field-sourced abalone parents. ‘Aquaculture’ family groups have ancestors sourced from Kangaroo Island and the Taylor’s Landing region, South Australia. Colours signify different family groups. “SS”,”FS” & ”ES” signify susceptible abalone from the Aquaculture, Farm Beach and Elliston populations, respectfully. “SR”,”FR” & ”ER” signify resilient abalone from the Aquaculture, Farm Beach and Elliston populations, respectfully.

.
