# Supplementary material for: Epipodial Tentacle Gene Expression and Predetermined Resilience to Summer Mortality in the Commercially Important Greenlip Abalone, Haliotis laevigata
Source: Mar Biotechnol (NY). 2017 Mar 27;19(2):191–205. doi: 10.1007/s10126-017-9742-z (PMC5405107; doi:10.1007/s10126-017-9742-z)
Supplement: Supplementary file 2 — (DOCX 78 kb) [file 10126_2017_9742_MOESM2_ESM.docx]

**Article Title:** Epipodial tentacle gene expression and predetermined resilience to summer mortality in the commercially important greenlip abalone, *Haliotis laevigata*

**Journal Name:** Marine Biotechnology

**Authors:**

Brett P. Shiel^1^, Nathan E. Hall^1,2,4^, Ira R. Cooke^3,2,4^, Nicholas A. Robinson^5,6^, Jan M. Strugnell^7,1^

1. Department of Ecology, Environment and Evolution, School of Life Sciences, La Trobe University, Kingsbury Drive, Melbourne, Vic. 3086, Australia
2. Life Sciences Computation Centre, VLSCI, Parkville, Vic, Australia
3. Department of Molecular and Cell Biology, James Cook University, Townsville, Australia
4. Department of Biochemistry, La Trobe Institute for Molecular Science, La Trobe University, Kingsbury Drive, Melbourne, Vic. 3086, Australia
5. Nofima, P.O. Box 210, 1431 Ås, Norway.
6. Sustainable Aquaculture Laboratory - Temperate and Tropical (SALTT), School of BioSciences, The University of Melbourne, Parkville, Vic. 3010, Australia
7. Centre for Sustainable Tropical Fisheries & Aquaculture, College of Science and Engineering, James Cook University, Townsville, Qld, 4811, Australia

**Corresponding author:**

Email: [bpshiel@students.latrobe.edu.au](mailto:bpshiel@students.latrobe.edu.au)

Phone: 0417151109

Fax: 03 9479 1266

Postal Address:

Brett Shiel

Department of Ecology, Environment & Evolution

La Trobe University, Plenty Road & Kingsbury Drive, Melbourne VIC 3086, Australia

Table S1. Top significant SwissProt hits for differentially expressed genes

| comp | Best.GeneNames | Best.GIs | Best.SPIDs | Best.EVALs | Description | Species |
| --- | --- | --- | --- | --- | --- | --- |
| comp100140_c0 | ZCWPW2 | 74740233 | ZCPW2_HUMAN | 4.00E-20 | Zinc finger CW-type PWWP domain protein 2 | *Homo sapien* |
| comp100812_c1 | yajO | 3915950 | YAJO_ECOLI | 5.00E-55 | Uncharacterized oxidoreductase YajO | Escherichia coli |
| comp101254_c0 | HSP70B2 | 1170377 | HSP74_ANOAL | 0.00E+00 | Heat shock protein 70 B2 | Anopheles albimanus |
| comp101317_c1 | slc6a9 | 190410910 | SC6A9_XENLA | 1.00E-148 | Sodium- and chloride-dependent glycine transporter 1 | Xenopus laevis |
| comp102187_c0 | Nwd1 | 172046158 | K1239_MOUSE | 5.00E-95 | NACHT and WD repeat domain-containing protein 2 | Mus musculus |
| comp102307_c1 | IIV6-193R | 33302608 | VF193_IIV6 | 4.00E-12 | Apoptosis inhibitor 193R | Invertebrate iridescent virus 6 (IIV-6) |
| comp102980_c0 | DNAH5 | 116241343 | DYH5_HUMAN | 0 | Dynein heavy chain 5, axonemal | Homo sapien |
| comp103803_c0 | pol | 110282984 | POL_FOAMV | 2.00E-06 | Pro-Pol polyprotein | Human spumaretrovirus (SFVcpz(hu)) |
| comp104314_c0 | SULT1C2 | 12229966 | ST1C2_RABIT | 4.00E-46 | Sulfotransferase 1C2 | Oryctolagus cuniculus |
| comp105782_c0 | UGT2C1 | 549160 | UD2C1_RABIT | 4.00E-93 | UDP-glucuronosyltransferase 2C1 | Oryctolagus cuniculus |
| comp107042_c0 | SAMD9 | 71153739 | SAMD9_HUMAN | 9.00E-89 | Sterile alpha motif domain-containing protein 9 | Homo sapien |
| comp107307_c0 | ANKRD65 | 363583286 | ANR65_HUMAN | 3.00E-13 | Ankyrin repeat domain-containing protein 65 | Homo sapien |
| comp107328_c0 | Mfn2 | 47605852 | MFN2_MOUSE | 0.00E+00 | Mitofusin-2 | Mus musculus |
| comp32926_c0 | CD109 | 117949389 | CD109_HUMAN | 1.00E-111 | CD109 antigen | Homo sapien |
| comp80721_c0 | DNAH5 | 116241343 | DYH5_HUMAN | 0 | Dynein heavy chain 5, axonemal | Homo sapien |
| comp84608_c0 | POT1 | 50400825 | POTE1_CHICK | 5.00E-48 | Protection of telomeres protein 1 | Gallus gallus |
| comp86148_c0 | TLR4 | 62903518 | TLR4_PIG | 3.00E-22 | Toll-like receptor 4 | Sus scrofa |
| comp86782_c0 | NA | 28201900 | STXA_SYNHO | 6.00E-08 | Stonustoxin subunit alpha | Synanceia horrida |
| comp87244_c1 | Ptprm | 341941771 | PTPRM_MOUSE | 9.00E-25 | Receptor-type tyrosine-protein phosphatase mu | Mus musculus |
| comp88885_c0 | RGS22 | 122064954 | RGS22_HUMAN | 6.00E-49 | Regulator of G-protein signaling 22; Short=RGS22 | Homo sapien |
| comp89220_c0 | ZNF99 | 205640301 | ZNF99_HUMAN | 2.00E-31 | Zinc finger protein 99 | Homo sapien |
| comp90034_c0 | ptp-3 | 29427539 | LAR_CAEEL | 7.00E-21 | Tyrosine-protein phosphatase Lar-like | Caenorhabditis elegans |
| comp93489_c0 | NA | 374110557 | ELDP1_LOTGI | 8.00E-09 | EGF-like domain-containing protein 1 | Lottia gigantea |
| comp94600_c0 | POLG | 2494178 | DPOG1_CHICK | 0.00E+00 | DNA polymerase subunit gamma-1 | Gallus gallus |
| comp95019_c0 | BLVRB | 1706870 | BLVRB_HUMAN | 3.00E-12 | Flavin reductase (NADPH) | Homo sapien |
| comp95089_c0 | RCN2 | 224487710 | CALU_RABIT | 8.00E-44 | Calumenin | Oryctolagus cuniculus |

Table S2. Top functional significant BlASTX hits to the oyster (*Crassostrea gigas*) genome for differentially expressed genes

| comp | Description | Max score | Total score | Query cover | E value | Ident | Accession |
| --- | --- | --- | --- | --- | --- | --- | --- |
| comp100140_c0 | PREDICTED: uncharacterized protein LOC105335639 isoform X1 [Crassostrea gigas] | 149 | 149 | 22% | 3.00E-38 | 37% | XP_011437922.1 |
| comp100812_c1 | PREDICTED: probable voltage-gated potassium channel subunit beta [Crassostrea gigas] | 394 | 394 | 62% | 7.00E-132 | 55% | XP_011450710.1 |
| comp101254_c0 | PREDICTED: heat shock protein 70 B2-like [Crassostrea gigas] | 1026 | 1026 | 81% | 0 | 79% | XP_011435905.1 |
| comp101317_c1 | PREDICTED: sodium- and chloride-dependent GABA transporter 1-like isoform X1 [Crassostrea gigas] | 92.4 | 92.4 | 77% | 4.00E-22 | 45% | XP_011434867.1 |
| comp102187_c0 | PREDICTED: NACHT and WD repeat domain-containing protein 1-like [Crassostrea gigas] | 862 | 1148 | 68% | 0 | 47% | XP_011444520.1 |
| comp102980_c0 | PREDICTED: dynein heavy chain 5, axonemal-like isoform X7 [Crassostrea gigas] | 2946 | 2946 | 77% | 0 | 85% | XP_011448836.1 |
| comp103803_c0 | PREDICTED: uncharacterized protein LOC105323737 isoform X1 [Crassostrea gigas] | 77.4 | 154 | 45% | 6.00E-32 | 40% | XP_011421129.1 |
| comp104314_c0 | PREDICTED: sulfotransferase family cytosolic 1B member 1-like [Crassostrea gigas] | 150 | 150 | 72% | 1.00E-42 | 39% | XP_011420695.1 |
| comp106323_c0 | PREDICTED: kyphoscoliosis peptidase-like isoform X1 [Crassostrea gigas] | 83.2 | 287 | 66% | 3.00E-16 | 53% | XP_011414010.1 |
| comp107042_c0 | PREDICTED: sterile alpha motif domain-containing protein 9-like [Crassostrea gigas] | 790 | 846 | 58% | 0 | 34% | XP_011448942.1 |
| comp107307_c0 | PREDICTED: uncharacterized protein LOC105330422 [Crassostrea gigas] | 45.1 | 45.1 | 15% | 7.00E-05 | 40% | XP_011430394.1 |
| comp107328_c0 | PREDICTED: mitofusin-2-like isoform X2 [Crassostrea gigas] | 89 | 89 | 9% | 3.00E-17 | 41% | XP_011449174.1 |
| comp165393_c0 | PREDICTED: uncharacterized protein LOC105322266 [Crassostrea gigas] | 102 | 193 | 73% | 1.00E-43 | 27% | XP_011419209.1 |
| comp32926_c0 | PREDICTED: CD109 antigen-like isoform X5 [Crassostrea gigas] | 644 | 644 | 91% | 0 | 63% | XP_011444700.1 |
| comp49337_c0 | PREDICTED: myosin heavy chain, non-muscle-like isoform X5 [Crassostrea gigas] | 52.4 | 52.4 | 53% | 3.00E-06 | 24% | XP_011440672.1 |
| comp80721_c0 | PREDICTED: dynein heavy chain 5, axonemal-like isoform X8 [Crassostrea gigas] | 1024 | 1024 | 99% | 0 | 77% | XP_011448838.1 |
| comp84608_c0 | PREDICTED: protection of telomeres protein 1-like [Crassostrea gigas] | 417 | 417 | 74% | 3.00E-134 | 38% | XP_011442438.1 |
| comp86148_c0 | PREDICTED: toll-like receptor 4 [Crassostrea gigas] | 134 | 134 | 85% | 5.00E-32 | 25% | XP_011422119.1 |
| comp87244_c1 | PREDICTED: receptor-type tyrosine-protein phosphatase alpha-like [Crassostrea gigas] | 117 | 169 | 99% | 1.00E-29 | 37% | XP_011455798.1 |
| comp88885_c0 | PREDICTED: regulator of G-protein signaling 22-like isoform X7 [Crassostrea gigas] | 227 | 227 | 26% | 2.00E-61 | 45% | XP_011413768.1 |
| comp89220_c0 | PREDICTED: zinc finger protein 37 homolog [Crassostrea gigas] | 306 | 306 | 40% | 9.00E-89 | 45% | XP_011452010.1 |
| comp92088_c0 | PREDICTED: uncharacterized protein LOC105345484 [Crassostrea gigas] | 63.9 | 63.9 | 46% | 1.00E-12 | 36% | XP_011451918.1 |
| comp94600_c0 | PREDICTED: DNA polymerase subunit gamma-1-like isoform X2 [Crassostrea gigas] | 1170 | 1170 | 82% | 0 | 54% | XP_011433469.1 |
| comp95019_c0 | PREDICTED: uncharacterized protein At2g34460, chloroplastic-like [Crassostrea gigas] | 187 | 187 | 24% | 1.00E-54 | 53% | XP_011435341.1 |
| comp95089_c0 | PREDICTED: calumenin-like isoform X2 [Crassostrea gigas] | 230 | 230 | 59% | 2.00E-70 | 44% | XP_011413831.1 |
| comp97949_c0 | PREDICTED: putative nuclease HARBI1 [Crassostrea gigas] | 66.6 | 153 | 21% | 1.00E-11 | 64% | XP_011432496.1 |

Table S3. Top BLASTn “somewhat similar sequence” search results for comp25540 and comp59699 differentially expressed genes

| comp | Description | Max score | Total score | Query cover | E value | Ident | Accession |
| --- | --- | --- | --- | --- | --- | --- | --- |
| comp25540_c0 | Haliotis diversicolor genomic DNA, BAC clone: 002_c14 | 50 | 50 | 3% | 0.11 | 91% | LC027314.1 |
| comp59699_c0 | Haliotis diversicolor genomic DNA, BAC clone: 006_rep_c2415 | 68 | 167 | 12% | 4.00E-07 | 71% | LC027343.1 |
