# Supplementary material for: Epipodial Tentacle Gene Expression and Predetermined Resilience to Summer Mortality in the Commercially Important Greenlip Abalone, Haliotis laevigata
Source: Mar Biotechnol (NY). 2017 Mar 27;19(2):191–205. doi: 10.1007/s10126-017-9742-z (PMC5405107; doi:10.1007/s10126-017-9742-z)
Supplement: Supplementary file 3 — (DOCX 42 kb) [file 10126_2017_9742_MOESM3_ESM.docx]

Reviewer Table 1. Comparison of the two differentially expressed genes between abalone from all locations. Gene expression results including all Elliston abalone (left) and results with Elliston Family 1 removed from the analysis (right).

| transcript | logFC | logCPM | LR | PValue | FDR | WithoutE1logFC | WithoutE1logCPM | WithoutE1LR | WithoutE1PValue | WithoutE1FDR |
| --- | --- | --- | --- | --- | --- | --- | --- | --- | --- | --- |
| comp25540_c0 | 5.409154 | -0.1096995 | 35.03553 | 3.24E-09 | 0.000112307 | 5.406233 | 0.03116194 | 36.63796 | 1.42E-09 | 4.88E-05 |
| comp59699_c0 | 5.277105 | 0.137134 | 27.63065 | 1.47E-07 | 0.002546829 | 5.26024 | 0.28151045 | 28.03837 | 1.19E-07 | 2.04E-03 |
