# Supplementary material for: Epipodial Tentacle Gene Expression and Predetermined Resilience to Summer Mortality in the Commercially Important Greenlip Abalone, Haliotis laevigata
Source: Mar Biotechnol (NY). 2017 Mar 27;19(2):191–205. doi: 10.1007/s10126-017-9742-z (PMC5405107; doi:10.1007/s10126-017-9742-z)
Supplement: Supplementary file 4 — (DOCX 133 kb) [file 10126_2017_9742_MOESM4_ESM.docx]

Reviewer Table 2. Comaprison of differential gene expression results between Elliston abalone. Gene expression results including all Elliston abalone (left) and results with Elliston Family 1 removed from the analysis (right).

| comps | ElogFC | ElogCPM | ELR | EPValue | EFDR | WithoutE1logFC | WithoutE1logCPM | WithoutE1LR | WithoutE1PValue | WithoutE1FDR |
| --- | --- | --- | --- | --- | --- | --- | --- | --- | --- | --- |
| comp107307_c0 | 4.684733124 | 5.301932144 | 45.82625371 | 1.29E-11 | 4.36E-07 | 4.675503396 | 5.287154281 | 47.14460057 | 6.59E-12 | 2.20E-07 |
| comp106927_c0 | 4.725340882 | 3.333579188 | 37.1269569 | 1.11E-09 | 1.87E-05 | 4.714917839 | 3.312612051 | 27.2770535 | 1.76E-07 | 0.0014704 |
| comp106512_c0 | -2.273734083 | 1.978333415 | 34.2097353 | 4.95E-09 | 5.57E-05 | -2.284275991 | 2.053220991 | 34.8904408 | 3.49E-09 | 5.82E-05 |
| comp106843_c1 | 9.204896595 | 2.860338272 | 27.35929659 | 1.69E-07 | 0.001426405 | 9.176568594 | 2.93852784 | 25.90635441 | 3.58E-07 | 0.002391376 |
| comp84649_c0 | 8.618175755 | 3.006781776 | 25.30542913 | 4.89E-07 | 0.002360735 | 8.592379039 | 3.073270843 | 29.63742012 | 5.21E-08 | 0.000579288 |
| comp100079_c0 | 6.634795266 | 2.541504627 | 25.47130618 | 4.49E-07 | 0.002360735 | 6.621785638 | 2.401170665 | 20.59423829 | 5.68E-06 | 0.01262605 |
| comp90034_c0 | 6.943211269 | 3.303678442 | 25.90619962 | 3.58E-07 | 0.002360735 | 6.929501437 | 3.235231444 | 20.29657703 | 6.63E-06 | 0.013828781 |
| comp84608_c0 | -1.52091936 | 3.032911037 | 23.66162494 | 1.15E-06 | 0.004673926 | -1.531560117 | 3.020825136 | 23.4622809 | 1.27E-06 | 0.007083351 |
| comp99055_c0 | -7.231557818 | 0.928552421 | 23.50544836 | 1.25E-06 | 0.004673926 | -7.213866852 | 0.951454753 | 21.10370006 | 4.35E-06 | 0.011533679 |
| comp92111_c0 | 4.326315682 | 2.067443623 | 23.26885366 | 1.41E-06 | 0.004757077 | 4.31377016 | 2.101219861 | 21.0416225 | 4.49E-06 | 0.011533679 |
| comp92088_c0 | -2.03459368 | 5.446908519 | 22.79540534 | 1.80E-06 | 0.005532181 | -2.046948242 | 5.534248476 | 22.20533058 | 2.45E-06 | 0.009527174 |
| comp102331_c0 | -3.015300812 | 1.707036914 | 22.4927856 | 2.11E-06 | 0.005936219 | -3.024184028 | 1.755542348 | 17.91939719 | 2.30E-05 | 0.027460165 |
| comp105540_c1 | 1.894779705 | 5.165869447 | 21.90845784 | 2.86E-06 | 0.00742886 | 1.884228233 | 5.128477773 | 19.88354843 | 8.23E-06 | 0.015709468 |
| comp100140_c0 | -2.659138186 | 2.681274256 | 21.59530462 | 3.37E-06 | 0.00779349 | -2.669301089 | 2.783014075 | 20.7900172 | 5.12E-06 | 0.0122131 |
| comp95078_c1 | 6.183911483 | 0.241571695 | 21.54200968 | 3.46E-06 | 0.00779349 | 6.155053258 | 0.15323908 | 18.7808661 | 1.47E-05 | 0.023295451 |
| comp80721_c0 | -2.365210541 | 0.356323962 | 20.76092765 | 5.20E-06 | 0.010982711 | -2.376244625 | 0.402843332 | 22.11343348 | 2.57E-06 | 0.009527174 |
| comp81142_c0 | -3.77076727 | 0.317201769 | 20.50804709 | 5.94E-06 | 0.011796226 | -3.777322584 | 0.407000503 | 21.5498817 | 3.45E-06 | 0.010879186 |
| comp104314_c0 | -1.843548057 | 2.788926823 | 20.26525504 | 6.74E-06 | 0.012647948 | -1.852748946 | 2.706450691 | 18.22666921 | 1.96E-05 | 0.026880273 |
| comp49337_c0 | -2.132176126 | 0.537336888 | 20.11924423 | 7.28E-06 | 0.012932671 | -2.143508189 | 0.596523846 | 19.11495482 | 1.23E-05 | 0.020531041 |
| comp106323_c0 | 7.419021285 | 5.841515601 | 19.98760164 | 7.79E-06 | 0.01316156 | 7.406401371 | 5.811270327 | 11.21044801 | 0.000813381 | **FDR>0.05** |
| comp90271_c0 | -3.888771947 | 2.003408009 | 19.69415479 | 9.09E-06 | 0.014614628 | -3.894514771 | 2.074898691 | 19.82747293 | 8.48E-06 | 0.015709468 |
| comp103081_c0 | 3.939032125 | 2.8735865 | 19.53803867 | 9.86E-06 | 0.015138046 | 3.927830713 | 2.848060893 | 15.22724653 | 9.53E-05 | **FDR>0.05** |
| comp103083_c0 | 7.112809316 | 3.044894701 | 19.23157202 | 1.16E-05 | 0.017000411 | 7.100347484 | 2.975923023 | 18.41619818 | 1.78E-05 | 0.02575367 |
| comp102187_c0 | 2.473168251 | 2.522200751 | 19.01639663 | 1.30E-05 | 0.01823634 | 2.462896192 | 2.446793215 | 22.75814402 | 1.84E-06 | 0.008756543 |
| comp95089_c0 | -1.696742017 | 2.635026189 | 18.81166687 | 1.44E-05 | 0.019490168 | -1.707536839 | 2.653234286 | 17.92836368 | 2.29E-05 | 0.027460165 |
| comp88885_c0 | 3.198478488 | 0.89825558 | 18.36022088 | 1.83E-05 | 0.022868661 | 3.186930887 | 0.968018853 | 17.25950804 | 3.26E-05 | 0.031995291 |
| comp105386_c1 | 2.879121167 | 3.616334815 | 18.36880827 | 1.82E-05 | 0.022868661 | 2.867566534 | 3.54587952 | 16.97227138 | 3.79E-05 | 0.034201351 |
| comp89194_c1 | -6.104514917 | 1.287527385 | 17.93480208 | 2.29E-05 | 0.027041804 | -6.117932018 | 1.451541751 | 21.47381207 | 3.59E-06 | 0.010879186 |
| comp84117_c0 | 7.641853681 | 1.712162295 | 17.84044779 | 2.40E-05 | 0.027041804 | 7.61609794 | 1.868804072 | 17.68032213 | 2.61E-05 | 0.029060952 |
| comp90629_c0 | 2.947482717 | 2.002046247 | 17.8692936 | 2.37E-05 | 0.027041804 | 2.936617876 | 2.095448016 | 16.7111919 | 4.35E-05 | 0.037261973 |
| comp89877_c0 | 6.281363653 | 0.560377574 | 17.70878781 | 2.57E-05 | 0.028044589 | 6.254310207 | 0.532530357 | 17.41705149 | 3.00E-05 | 0.031290482 |
| comp102980_c0 | -2.068014706 | 2.850978526 | 17.51059254 | 2.86E-05 | 0.028779062 | -2.079006004 | 2.889010205 | 17.51186954 | 2.86E-05 | 0.030728357 |
| comp89220_c0 | -1.415505931 | 1.391338196 | 17.55046617 | 2.80E-05 | 0.028779062 | -1.425415345 | 1.401334063 | 17.33937621 | 3.13E-05 | 0.031608016 |
| comp70923_c0 | 2.386876211 | 1.538258934 | 17.48395862 | 2.90E-05 | 0.028779062 | 2.375456961 | 1.518545489 | 17.09988482 | 3.55E-05 | 0.033805732 |
| comp74664_c0 | -6.499876199 | 0.301594797 | 17.32057178 | 3.16E-05 | 0.029619988 | -6.482723872 | 0.463205464 | 16.98667556 | 3.76E-05 | 0.034201351 |
| comp87244_c1 | 5.208115897 | 2.807832288 | 17.35664693 | 3.10E-05 | 0.029619988 | 5.19692715 | 2.801296155 | 12.65545642 | 0.000374471 | **FDR>0.05** |
| comp94600_c0 | -1.350597625 | 2.595508198 | 17.14320484 | 3.47E-05 | 0.030806911 | -1.361341582 | 2.632035767 | 18.15934973 | 2.03E-05 | 0.026880273 |
| comp103083_c2 | 5.857895469 | 3.172755564 | 17.17931341 | 3.40E-05 | 0.030806911 | 5.848216692 | 3.110452013 | 16.67162032 | 4.44E-05 | 0.037261973 |
| comp95019_c0 | 2.040348764 | 3.962223205 | 16.92113929 | 3.90E-05 | 0.03374089 | 2.02936597 | 3.959611326 | 11.21579828 | 0.000811039 | **FDR>0.05** |
| comp93725_c0 | 8.136252866 | 2.716538886 | 16.7870498 | 4.18E-05 | 0.035305652 | 8.106550416 | 2.672648399 | 15.81265128 | 6.99E-05 | 0.04761604 |
| comp106120_c0 | -2.848971097 | 1.787894936 | 16.6421255 | 4.51E-05 | 0.036293617 | -2.860936186 | 1.916358109 | 16.01937971 | 6.27E-05 | 0.045284667 |
| comp86148_c0 | -3.034191908 | 0.138651065 | 16.65371787 | 4.49E-05 | 0.036293617 | -3.0403541 | 0.223191636 | 14.2842304 | 0.000157176 | **FDR>0.05** |
| comp78364_c0 | -2.120290787 | 0.325525094 | 16.46435875 | 4.96E-05 | 0.038362445 | -2.130122983 | 0.33586755 | 18.64505985 | 1.57E-05 | 0.02387801 |
| comp105782_c0 | 1.794564946 | 2.901442985 | 16.44878668 | 5.00E-05 | 0.038362445 | 1.784948151 | 2.890194524 | 15.44339664 | 8.50E-05 | **FDR>0.05** |
| comp95938_c0 | -3.427991542 | 0.222901217 | 16.39791876 | 5.13E-05 | 0.038530138 | -3.434431537 | 0.243304045 | 15.3709149 | 8.83E-05 | **FDR>0.05** |
| comp103803_c0 | 7.105085447 | 1.327887214 | 16.1197673 | 5.95E-05 | 0.043652808 | 7.076921284 | 1.221964298 | 16.30428397 | 5.39E-05 | 0.042849105 |
| comp92494_c0 | 2.076870706 | 1.73490961 | 16.0183151 | 6.27E-05 | 0.04507543 | 2.066268945 | 1.714942763 | 16.00694227 | 6.31E-05 | 0.045284667 |
| comp84161_c1 | 5.919574256 | 0.690939365 | 15.93434739 | 6.56E-05 | 0.046138094 | 5.892209686 | 0.638674944 | 15.9227873 | 6.60E-05 | 0.045859913 |
| comp78179_c0 | -6.440442025 | 0.175898534 | 15.87967466 | 6.75E-05 | 0.046521104 | -6.422764847 | 0.335044857 | 15.29341222 | 9.20E-05 | **FDR>0.05** |
| comp24011_c0 | -4.471643845 | 0.284241637 | 15.83869889 | 6.90E-05 | 0.046588614 | -4.470243674 | 0.418348789 | 11.75422854 | 0.000607052 | **FDR>0.05** |
| comp97812_c0 | 3.776047364 | 3.583839917 | 15.51820179 | 8.17E-05 | 0.04754908 | 3.766587324 | 3.63851336 | 18.10110621 | 2.09E-05 | 0.026880273 |
| comp101317_c1 | 4.636262505 | 3.691771279 | 15.55087566 | 8.03E-05 | 0.04754908 | 4.623881784 | 3.712919411 | 16.09707141 | 6.02E-05 | 0.045284667 |
| comp106153_c0 | -2.73858776 | 2.343003167 | 15.6189514 | 7.75E-05 | 0.04754908 | -2.750776924 | 2.452539189 | 15.26299763 | 9.35E-05 | **FDR>0.05** |
| comp107328_c0 | 1.932047936 | 4.258892769 | 15.58021879 | 7.91E-05 | 0.04754908 | 1.921315099 | 4.271284765 | 14.45172981 | 0.000143798 | **FDR>0.05** |
| comp100812_c1 | 3.039271785 | 3.45263211 | 15.48707954 | 8.31E-05 | 0.04754908 | 3.030466758 | 3.399747192 | 14.20013057 | 0.000164359 | **FDR>0.05** |
| comp32926_c0 | -2.260585122 | 0.038259503 | 15.59591004 | 7.84E-05 | 0.04754908 | -2.266780645 | 0.022470309 | 13.97968123 | 0.000184797 | **FDR>0.05** |
| comp97949_c0 | 9.809786986 | 1.686960055 | 15.64284019 | 7.65E-05 | 0.04754908 | 9.782349036 | 1.843326316 | 13.96519303 | 0.000186227 | **FDR>0.05** |
| comp46150_c0 | 3.759029163 | 2.226211586 | 15.70930247 | 7.39E-05 | 0.04754908 | 3.747884706 | 2.227178742 | 11.4958695 | 0.00069751 | **FDR>0.05** |
| comp90466_c0 | 4.334070968 | 1.490228441 | 15.67816638 | 7.51E-05 | 0.04754908 | 4.320900734 | 1.461552027 | 10.88597947 | 0.000968949 | **FDR>0.05** |
| comp86348_c0 | 4.9345825 | 2.277851291 | 15.27227947 | 9.31E-05 | **FDR>0.05** | 4.926824422 | 2.185824329 | 19.55304098 | 9.78E-06 | 0.017181043 |
| comp62124_c0 | 4.917511301 | 1.94229438 | 15.2092261 | 9.62E-05 | **FDR>0.05** | 4.905258658 | 1.976040405 | 17.73167842 | 2.54E-05 | 0.029060952 |
| comp99050_c0 | -3.320200358 | 1.351245851 | 14.9869332 | 0.000108258 | **FDR>0.05** | -3.334550553 | 1.475809356 | 16.66167026 | 4.47E-05 | 0.037261973 |
| comp104203_c0 | 2.774562583 | 3.032356862 | 14.67252959 | 0.000127897 | **FDR>0.05** | 2.76384095 | 3.026681796 | 16.42222744 | 5.07E-05 | 0.041245914 |
| comp91518_c0 | 2.436397108 | 1.314933037 | 14.34019936 | 0.000152572 | **FDR>0.05** | 2.424281955 | 1.419320536 | 16.02458896 | 6.25E-05 | 0.045284667 |
| comp93741_c0 | -2.190414668 | 1.680692814 | 12.31481203 | 0.000449378 | **FDR>0.05** | -2.203670157 | 1.75072684 | 15.98653505 | 6.38E-05 | 0.045284667 |
| comp103201_c3 | 6.656059621 | 1.05094137 | 14.7239172 | 0.000124457 | **FDR>0.05** | 6.626951622 | 1.075041436 | 15.70537488 | 7.40E-05 | 0.049386282 |
